# Comparison of statistical modelling methods for population-level gestational weight gain trajectories in ethnically diverse women in southeast Melbourne, Australia

**DOI:** 10.1136/bmjopen-2024-088664

**Published:** 2025-03-13

**Authors:** Sanjeeva Ranasinha, Helena J Teede, Cheryce Harrison, Rui Wang, Joanne Enticott

**Affiliations:** 1 Monash Centre for Health Research & Implementation, Monash University, Melbourne, Victoria, Australia; 2 Faculty of Medicine, Nursing & Health Sciences, Monash University, Melbourne, Victoria, Australia; 3 NHMRC Clinical Trials Centre, The University of Sydney, Sydney, New South Wales, Australia; 4 Department of Obstetrics and Gynaecology, Monash University, Clayton, Victoria, Australia

**Keywords:** Public health, Methods, STATISTICS & RESEARCH METHODS

## Abstract

**Abstract:**

**Objectives:**

Adverse lifestyle promotes escalating excess gestational weight gain (GWG) driving poor maternal and neonatal health outcomes. Recommended pregnancy lifestyle interventions rely on accurate assessment and prediction of GWG. A modelling technique to accommodate the complexities of GWG data and allow for the inclusion of maternal factors that influence the variation in GWG trajectory across pregnancy is necessary. We aimed to explore and determine the optimal statistical methods to accommodate data complexities such as nonlinearity, skewness and kurtosis and to model GWG trajectories from a large dataset of ethnically diverse pregnant women.

**Design and setting:**

This is a retrospective, observational study of routinely collected health data from women with singleton pregnancies from 2017 to 2021 delivering at one of the largest hospital networks in Australia, located in southeast Melbourne.

**Participants:**

There were 39 846 women with singleton pregnancies. Women had measurements taken during routine care at several time points throughout the pregnancy. Participants were from a diverse ethnic population, with the majority born overseas from 136 different countries (grouped into 12 world regions).

**Outcome:**

GWG was defined as the weight measured minus pre-pregnancy weight. Multiple statistical approaches were applied to model GWG trajectories: linear regression, cubic polynomial, neural network, generalised linear models and general additive model for location, scale and shape (GAMLSS) Box-Cox suite of models (including fitting fractional polynomials, cubic splines and penalised B-splines).

**Results:**

The dataset included 39 846 women and 109 339 GWG measurements. The two best-fitting models were derived using the GAMLSS Box-Cox t distribution: one with penalised B-splines and the other with cubic splines. Both models yielded the lowest Akaike information criterion and a generalised R-squared of 0.70. However, residual analysis indicated a preference for the model with penalised B-splines, making it the optimal choice. Using this optimal model, we demonstrate how to generate centile charts for the sample population.

**Conclusion:**

The optimal model developed will underpin our new epidemiological tool for the assessment and prediction of GWG. Using the model, individualised centile charts are relatively easy to produce, making them accessible to both healthcare providers and pregnant individuals. The visual nature of centile graphs makes it easier to see whether a woman’s GWG is on track, which is helpful for making informed decisions about nutrition, lifestyle and healthcare.

**Strengths and limitations of this study**

- This study has several strengths, including a diverse cohort across multiple large-scale maternity hospitals, and detailed capture of gestational weight gain (GWG) during pregnancy. As a general population-based cohort study, it provides a good representation of different ethnic groups and a fair representation of the prevalence of inadequate and excess GWG.
- All potential models were tested to derive the best-fitting model to accommodate the complexity of GWG.
- This is the primary step in building a GWG calculator that could be used by health professionals and consumers.
- Limitations include that categorising ancestry is challenging, with no agreed global approach. Classifying ethnicity by the country of birth may have introduced some inaccuracies; therefore, we aim to improve on this by exploring self-identified ethnicity in the future.
- Although clinical measurements of height and weight are considered the gold standard, self-reported pre-pregnancy measurements were shown to be a reasonable proxy and are also used widely in the literature.

## Introduction

 Healthy gestational weight gain (GWG) is important, with GWG recommendations well-established and clearly associated with better maternal and neonatal outcomes.^1 2^ Likewise, lifestyle interventions that optimise healthy GWG have been shown to improve health outcomes.^3 4^ However, there is a lack of sophisticated statistical models and pregnancy weight centile charts and tools to guide both health professionals and women on assessing and achieving healthy GWG. This fundamental gap significantly curtails the ability to provide an accurate, prospective estimation of personalised GWG trajectories to optimise pregnancy outcomes aligned with international GWG recommendations.^5^

As GWG is a critical factor in maternal and fetal health, the Institute of Medicine (IOM) has provided guidelines based on pre-pregnancy body mass index (BMI).^1^ These BMI guides are based on the WHO BMI classifications, which were primarily derived from studies in predominantly white, Western populations.^1^ BMI categories can be different for different ethnic groups; and in the published literature are BMI categories for Chinese and Korean populations, with more ethic-specific data forthcoming.^26^,^8^ GWG deviations from the IOM recommendations—either excessive or inadequate weight gain—are associated with adverse outcomes.^1^,^3^ Excessive GWG increases the risk of gestational diabetes, hypertensive disorders, caesarean delivery and childhood obesity, while inadequate GWG raises the likelihood of preterm birth, low birth weight and fetal growth restriction.^1^,^3^ IOM recommends that monitoring and individualised guidance are essential to optimise maternal and neonatal outcomes.^1^,^3^

Multiple challenges exist in advising women on healthy GWG during pregnancy.^9^ First, accurate assessment of BMI at conception is challenging as many women are not weighed until well into their pregnancy when they attend for antenatal care, yet estimation of BMI is important to inform prospective healthy GWG recommendations. Women with lower BMI have greater recommended GWG, and those with a higher BMI have less recommended GWG to achieve optimal health outcomes.^1 10^ Second, healthy GWG itself is currently provided as retrospective total recommended weight gain over the course of pregnancy, which can make the advice for individual women during the pregnancy more difficult.^1^ Third, multiple factors may influence individual GWG, including BMI at conception, ethnicity, parity and maternal age and are often not considered. Furthermore, GWG trajectories are not linear during pregnancy. Currently, there are no statistically driven approaches to inform individualised and differential healthy rates of GWG. Hence, GWG recommendations are difficult to personalise and monitor in real time during a pregnancy.

Apart from nonlinearity, there are multiple complexities with GWG data including a high likelihood for the data to be skewed (asymmetrical distribution with a long tail on the right side) and kurtotic (extreme values with light tails). Current literature has failed to factor in the variability of GWG trajectories across pregnancy, the multiple influencing factors and the data complexity. Comparison with previous studies on GWG is difficult because of variations in study design, purpose and methods. Some studies are limited in diversity in the study population, hence lacking generalisability, and most studies do not evaluate multiple appropriate modelling techniques to determine the best statistical method in estimating GWG. The association of factors such as ethnicity, parity and GWG are not included in these studies. Hence, optimal methods to monitor and guide clinical recommendations for GWG in an individual woman remain unclear.

Here, we aimed to use routine clinical data from a large diverse population of pregnant women to:

1. Assess the reliability of the pre-pregnancy weight and BMI recorded at the health service.
2. Explore and compare multiple potential candidate statistical models to determine the optimal method to model GWG trajectories in women as they progress through pregnancy.
3. Demonstrate the feasibility of producing GWG trajectory centile graphs from the optimal statistical model.

## Methods

### Study design and setting

This retrospective longitudinal observational study involved the secondary analysis of patient data routinely collected by the Monash Health Network between 2017 and 2021. We used individual participant data from the largest antenatal health service in Australia with multiple maternity hospitals and a diverse ethnic and socioeconomic population, in the context of a free universal healthcare system. The Birthing Outcomes System (BOS) electronic database was used to derive demographic (age, ethnicity and parity), obstetric history and pregnancy outcome data on all singleton pregnancies routinely collected for the duration of the pregnancy. This study is reported following the Reporting of studies Conducted using Observational Routinely collected health Data (RECORD) guidelines,^11^ and the RECORD checklist is found in online supplemental table S7. The number of women by visit and gestational age is shown in online supplemental table S1.

### Participants

Individual participant data from singleton pregnancies from Monash Health multiple hospital services from BOS were used in this study. Women with multiple pregnancies were excluded because the GWG trajectory is expected to be different from singleton pregnancies. Women had measurements taken during routine care at several time points throughout the pregnancy. Participant data were de-identified, and consent was not directly obtained. The project was approved by the Monash Health Human Research Ethics Committee (project no. RES-21-0000183 L).

### Patient and public involvement

Patients who provided data were not involved in setting the research aims, but the organisation made them aware that their data may be used in research. Public involvement on the issue of GWG and healthy lifestyle in pregnancy by our team has been extensive. Gaps and needs were identified including co-design of an online tool to enable them to track and monitor healthy GWG during pregnancy (with the optimal model identified in this study as the underlying algorithm).

### Statistical analysis

Maternal pre-pregnancy BMI and age were continuous variables. Ethnicity was determined by country of birth, and we categorised ethnicity into 12 regions defined by the Australian Bureau of Statistics (online supplemental table S5). Parity was captured in three categories (0, 1–3, >3). They were time-fixed variables. Gestational age and GWG were continuous variables and varied with time.

Maternal height and weight were self-reported at remote booking visits in early pregnancy and were directly measured at the first antenatal visit. Maternal pre-pregnancy BMI was categorised as underweight (<18.5 kg/m^2^), normal weight (18.5–24.9 kg/m^2^), overweight (25.0–29.9 kg/m^2^) and obese (≥30 kg/m^2^) according to the WHO criteria. We used ethnic-specific BMI categories for Chinese and Korean BMI categories (China: underweight BMI <18.5 kg/m^2^, normal weight 18.5–23.9 kg/m^2^, overweight 24–28 kg/m^2^ and obese ≥28 kg/m^2^; Korea: underweight BMI <18.5 kg/m^2^, normal weight 18.5–22.9 kg/m^2^, overweight 23–25 kg/m^2^ and obese ≥25 kg/m^2^), while Japanese and Taiwanese studies used WHO BMI categories (underweight <18.5 kg/m^2^, normal weight 18.5–24.9 kg/m^2^, overweight 25–29.9 kg/m^2^ and obese ≥30 kg/m^2^).

The weight data were obtained from the electronic data captured into the hospitals’ computerised system at each visit. Weight data were recorded from week 4 onwards. GWG was calculated as the difference between the measured weight at a certain gestational age and the pre-pregnancy weight. The baby’s birth weight was obtained from routine clinical data, using a calibrated electronic scale.

Excluded from the modelling were GWG data points outside 4 SD in order to restrict weight gain to a credible interval. Imputation of missing values was not conducted. We modelled GWG from week 0 onwards. We did not assign a weight gain of 0 kg at the start of pregnancy (0 weeks) as it would have led to computational difficulties due to the lack of variation. Therefore, we used the measurement error of body weight of 0.70 kg, previously reported in a study that compares the accuracy of the mean weight registered by home scales.^12 13^ The variance of the gain score at week 0 was then equal to 0.70^2^+0.70^2^ = 0.98 kg. For each woman, the weight gain at 0 weeks was a random sample drawn from the normal (Gaussian) distribution with a mean of 0 and a variance of 0.98 kg.^13^ Allowing weight gain at the start of the pregnancy to vary partially captures the measurement error induced by self-reported pre-pregnancy weight. The starting values (0 weeks) have little bearing on the subsequent weeks and could be considered a nudge to initiate the iterations.^13 14^

#### Baseline weight reliability: aim 1

To assess the reliability of baseline weight, we used two-way mixed-effects models to calculate the intraclass correlation coefficient using an absolute agreement definition and the consistency of agreement. This means to evaluate whether the two data points (self-reported weight at the start of pregnancy and weight at the first measurement) match each other exactly. If they do not match exactly, we also check if one measurement is consistently higher or lower than the other.

#### Optimal GWG modelling: aim 2

Our aim was to determine the optimal method to model GWG trajectories in women across gestational age, adjusting for significant independent variables listed above that are known to influence GWG trajectories. As GWG trajectories by gestation time appeared nonlinear, we examined several statistical modelling approaches to determine which method fits GWG data the best.^15^ Multiple models were examined (seven in total), and for brevity only four of these will be included in detail in this manuscript (others included in online supplemental file). Three of these models will be the best-performing models, and the fourth model will be the linear regression model as a baseline model for comparison and to facilitate the interpretation of the coefficients (table 2). The three best-performing models were cubic polynomial and two generalised additive models (GAMs) for location, scale and shape (GAMLSS) Box-Cox model with original t distribution and with different smoothing functions (one with cubic splines^16^ and the other with penalised B-splines).^17 18^

The linear regression model examined the relationship between GWG by gestation week, assuming a linear association between them. Cubic polynomial models are used to capture nonlinear relationships between variables by including terms up to the third degree, providing flexibility in modelling trends or patterns in the data. We examined a number of these, and the main paper includes the model with GWG as a linear term, a squared term and a cubic term, and the other independent variables as linear or categorical.

The GAMLSS statistical framework enables flexible regression and smoothing models to be fitted to the data. The GAMLSS model assumes that the dependent variable has any parametric distribution which might be heavy- or light-tailed, and positively or negatively skewed. The Box-Cox original t distribution is ideal for handling skewed data and data with heavy tails or outliers due to its incorporation of a t distribution. Furthermore, all the parameters of the distribution (location, scale and shape) can be modelled as smooth functions of explanatory variables.^13 14^ We used the GAMLSS package in R V.4.1.2.^19^

To evaluate model performance, we compared model indices and residual statistics by visual inspection of quantile-quantile plots of the residuals, plots of residuals versus fitted values, detrended quantile-quantile plots and worm plots. Goodness-of-fit was evaluated using Akaike information criteria (AIC) and Schwarz/Bayesian information criteria (SBC).

**Figure 1:**
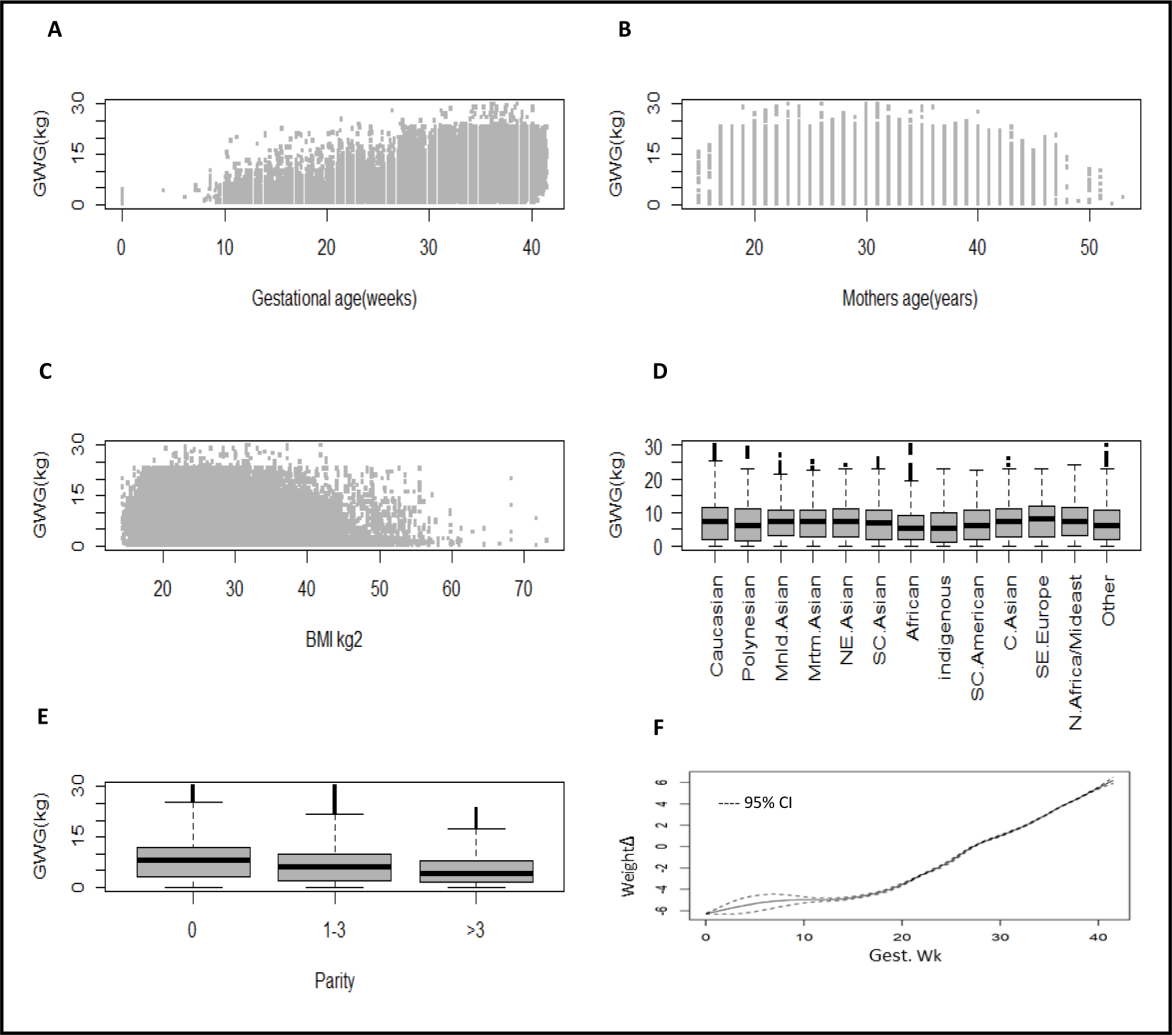
Plot of the GWG (kg) against explanatory variables gestational age (A), mothers’ age (B), BMI (C), ethnicity (D), parity (E) and the smoothed GWG (Δ) by gestational age (weeks) from the univariate GAM (F). BMI, body mass index; GAM, generalised additive model; GWG, gestational weight gain.

In brief, the other models examined were generalised linear model (GLM), cubic polynomial, GAMs and GAMLSS with smoothing function of fractional polynomials^19^ (table 2, figure 1 and online supplemental figure S1). We also examined a neural network method (online supplemental figure S2) to preliminarily explore if a machine learning technique could outperform conventional methods. For the GAMLSS models, we also examined another three distributions: normal, Box-Cox Cole-Green original and Box-Cox Power Exponential original. See online supplemental tables S2–S4 and online supplemental figures S1 and S3–S5.

All variables included in the model were described above.

#### Feasibility of GWG trajectory centile graphs: aim 3

Using only the optimal model, we will produce visual outputs of the GWG trajectories as centile charts using the data from the entire dataset and also for the Caucasian ethnicity classified by BMI. The lines on these charts will show the GWG centiles weight gain trajectories for gestational age: ie, (P2.3 (−2 SD), P16 (−1 SD), P50 (0 SD), P84 (1 SD) and P97.7 (2 SD).

We will also generate four separate trajectory centile charts, one for each of the pre-pregnancy BMI categories. These latter outputs were requested by our clinician stakeholders because the different BMI categories are known to be associated with different clinical outcomes.

### Sensitivity analysis

We compared the trajectories determined from the optimal modelling technique using a slightly smaller dataset by omitting the pre-pregnancy self-reported weight. Measured weight at the first visit becomes the baseline (instead of the self-reported pre-pregnancy weight), and GWG trajectories are calculated using the optimal modelling approach determined earlier. This sensitivity analysis is conducted to investigate the level of concordance in GWG trajectories between the two primary weight measurements.

## Results

Data included 39 846 women, with 109 339 GWG measurements. The average number of GWG measurements for each woman was 2.85 (min=1 and max=21). Table 1 shows the characteristics of the pregnant sample (n=39 846). Participants were from a diverse ethnic population, with the majority born overseas from 136 different countries (grouped into 12 world regions).

**Table 1:** Characteristics of the participating pregnancy cohorts (n=39 846)

Age (mean, SD)	28.4 (4.86)
Ethnicity no. (%)	
Australian/European	16 503 (41.4)
Polynesian	611 (1.5)
Mainland Asian	2945 (7.4)
Maritime Asian	1595 (4.0)
Northeast Asian	1953 (4.9)
South central Asian	9073 (22.8)
African	1327 (3.3)
Indigenous/Torres Strait Island	288 (0.7)
South America	205 (0.5)
Southeast Europe	2970 (7.5)
Eastern Europe	1198 (3.0)
North Africa and the Middle East	509 (1.3)
Other*	669 (1.7)
Gestation interval—median weight (kg) IQR	
1–9.9	71 (63.5–80)
10–15.9	66 (58–77)
16–20.9	66.5 (59–77)
21–25.9	69.5 (61–80)
26–30.9	72 (64–83)
31–35.9	75 (66.5–85.5)
36–40.9	76 (68–86.5)
≥41	79 (71–89)
Parity no. (%)	
0	16 060 (40.3)
1–4	22 276 (56.0)
>3	1510 (3.8)
Maternal complications no. (%)	
Gestational diabetes—diet control	266 (1.78)
Gestational diabetes—insulin-dependent	1248 (7.9)
Pre-existing diabetes type 2—insulin-dependent	113 (0.71)
Pre-existing diabetes—type 1	41 (0.26)
Pre-eclampsia—mild	1090 (6.89)
Pre-eclampsia—severe	75 (0.47)
Pregnancy-induced hypertension	408 (2.58)
Gestation weeks (mean, SD)	38.9 (1.71)

* Other includes Bahamas, Brunei, Darussalam, Dominican Republic, East Timor, Equatorial Guinea, Israel, Lesotho, Malta, Not Stated, South Africa and not- stated.

### Aim 1: baseline weight reliability and assessment

**Figure 2:**
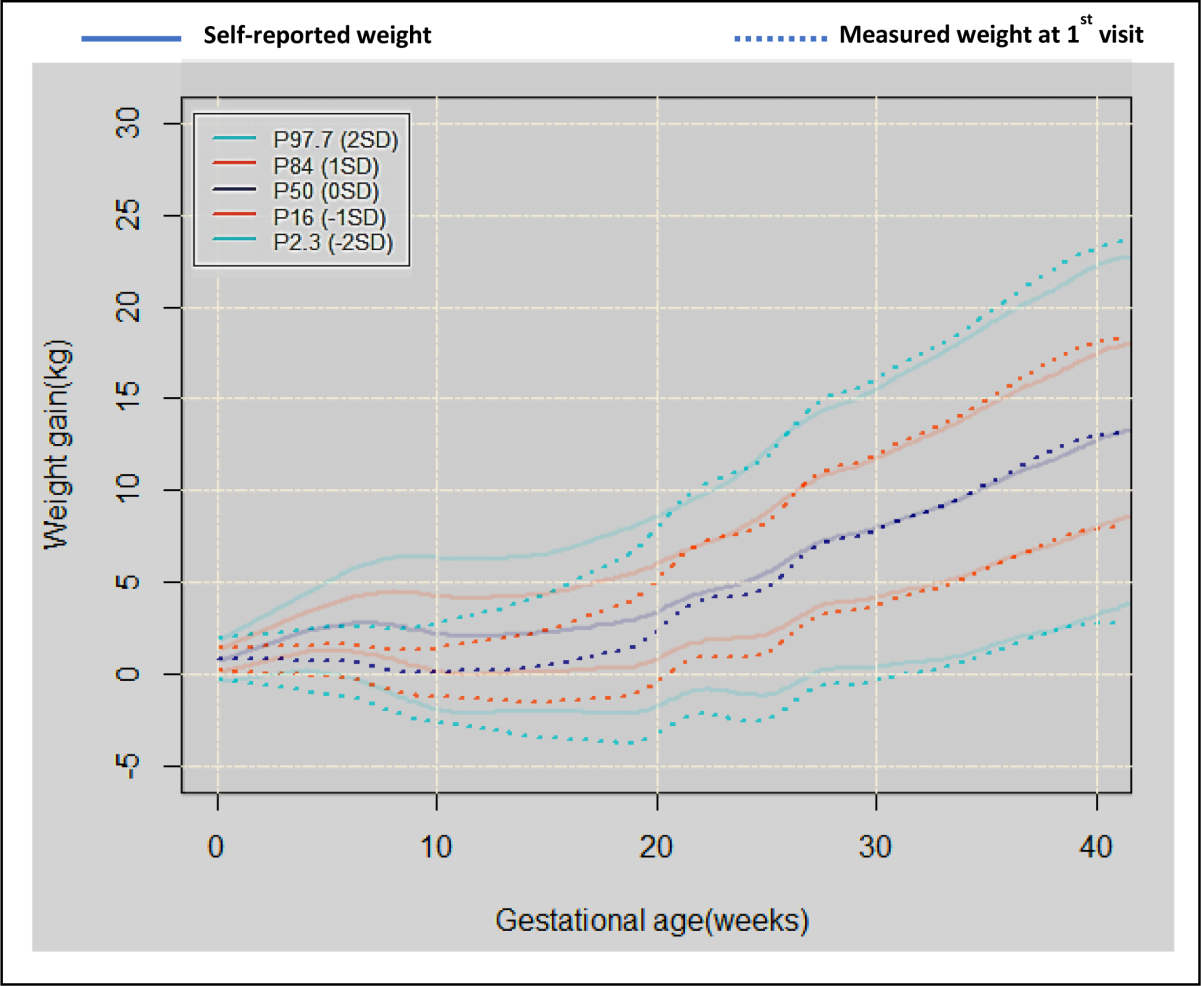
GWG trajectories generated when using the self-reported pre-pregnancy weight at week 0 (solid lines). Dotted lines show the GWG trajectories generated when the measured weight at the first visit is used as the week 0 weight instead. To avoid a ‘0’ GWG value at week 0, all week 0 GWG distributions were estimated using a Gaussian distribution. This can be considered a nudge to initiate the model iterations which have no bearing on the trajectories estimated with the measured weights. The measurement error of a single dial measurement is about 0.70 kg, so the variance of the gain score is 0.702+0.702 = 0.98 kg. For each woman, the weight gain at the start of pregnancy was taken as a random draw from the Gaussian distribution with a mean of 0 and a variance of 0.98 kg. The size of the measurement error was used since it is theoretically based, but any variance could have been applied. GWG, gestational weight gain.

An analysis conducted to assess the correlation between self-reported weight at the start of pregnancy and weight at first measurement 4–10 weeks using a two-way mixed-effects model revealed that the intraclass correlation coefficient using an absolute agreement definition was 0.98% (95% CI 86% to 0.99%). The consistency of agreement was 0.99 (95% CI 0.92 to 0.99). This informed the weight and BMI to be used in the GWG model, with both self-reported and weight at first measurement considered to be reliable. Figure 2 shows the GWG trajectories using the optimal model both with and without the baseline weight measurement (described in the Sensitivity analysis section).

### Aim 2: optimal GWG modelling

Characteristics of the Monash Health birthing outcome database are given in table 1. The number of women per gestational age and visit is given in online supplemental table S1. Figure 1 shows the plots of GWG against each of the explanatory variables and the smoothed relationship between GWG and gestational age. Although these are bivariable exploratory plots and take no account of the potential interaction between the explanatory variables, they indicate the complexity of GWG data. The first three explanatory variables, namely, gestational age, mothers’ age and BMI are continuous. The plot of GWG against gestational age suggests a positive relationship with an increased variation for higher gestation weeks. The assumption of homogeneity in the variance of the GWG appears to be violated here. There is also some indication of positive skewness in the distribution of the GWG against BMI. The box and whisker plots display how the GWG varies according to the explanatory factors’ ethnicity and parity. The median GWG decreases with increasing parity. Skewness is evident for ethnicity with nonsymmetrical boxes about the median and longer upper than lower whiskers for some ethnicities. The remaining plot (F) shows the nonlinear relationship between GWG and gestational age.

Overall, the two GAMLSS Box-Cox models in table 2 were assessed as the best-performing models, and the optimal model was the Box-Cox t distribution with penalised B-spline model. Both models produced the lowest AIC and the highest R-squared values (0.70), and the residual analysis (online supplemental figure S1) favoured the Box-Cox t distribution with penalised b-spline model. All terms contributed significantly to modelling the predictor GWG. The residual statistics are under the summary of quantile residuals. The (normalised quantile) residuals of the optimal model behave well, with the mean close to 0, variance close to 1, coefficient of skewness near 0, coefficient of kurtosis near 3 and the Filliben correlation coefficient (coefficient correlation test for normality) close to 1. One of the properties of the fitted nonparametric smooth functions is that they cannot be described in a mathematical form such as parametric terms.^14^ However, they can be displayed as a term plot (online supplemental figure S6). The plot shows that weight gain, gestation age and BMI have a nonlinear trend. For the factor variable parity, GWG is the highest for the nulliparous and tends to decrease depending on the number of offsprings for multiparous women. The shaded areas are the point-wise confidence bands for the smoothing curves and factor levels. These plots show that the optimal model allows for a flexible specification of the dependence of the parameter predictors on different explanatory terms in this application.

**Table 2:** Model comparisons

Parameters	Linear regression				GAMLSS (cubic spline)†			
	Estimate	95% CI		Significance	Estimate	95% CI		Significance
Intercept	3.936	3.75	4.12	***	0.161	0.131	0.19	***
Gestational age (weeks)	0.29	0.28	0.29	***	0.081	0.081	0.082	***
Gestational age^2^								
Gestational age^3^	–	–	–		–	–	–	
Mothers’ age (years)	−0.016	−0.02	−0.01	***	−0.002	−0.003	−0.001	***
BMI, kg/m^2^	−0.106	−0.11	−0.1	***	−0.02	−0.021	−0.019	***
Ethnicity								
Caucasian	Reference							
Polynesian	0.254	0.05	0.46	*	0.03	−0.002	0.063	
Mainland Asian	−1.11	−1.2	−1.03	***	−0.131	−0.142	−0.12	***
Maritime Asian	−0.788	−0.9	−0.67	***	−0.1	−0.116	−0.085	***
Chinese Asian	−0.7	−0.81	−0.59	***	−0.088	−0.102	−0.075	***
Southeast Asian	−0.859	−0.92	−0.8	***	−0.11	−0.119	−0.102	***
African	−1.483	−1.62	−1.35	***	−0.226	−0.247	−0.205	***
Indigenous/Torres Strait Island	−0.473	−0.78	−0.17	**	−0.107	−0.161	−0.053	***
South America	−0.336	−0.66	−0.02	*	−0.04	−0.085	0.005	
Southeast Europe	−0.677	−0.76	−0.59	***	−0.078	−0.089	−0.067	***
Eastern Europe	0.405	0.27	0.54	***	0.046	0.028	0.064	***
North Africa and the Middle East	−0.211	−0.37	−0.05	*	−0.016	−0.039	0.006	
Other	−0.086	−0.36	0.19		−0.033	−0.07	0.005	
Parity	Reference							
0								
1–3	−0.717	−0.77	−0.67	***	−0.09	−0.097	−0.083	***
>3	−1.532	−1.68	−1.39	***	−0.249	−0.275	−0.223	***
Goodness-of-fit statistics								
AIC	556 905				475 050			
SBC	557 087				476 080			
R^2^	0.53				0.70‡			
Summary of the quantile residuals								
Mean	2.00E-16				−0.005			
Variance	1				0.98			
Coefficient of skewness	0.51				0.055			
Coefficient of kurtosis	3.8				2.8			
Filliben correlation coefficient	0.991				0.9996			

**Parameters**	**Cubic polynomial**				**GAMLSS (penalised B-spline)†**			
	Estimate	95% CI		Significance	Estimate	95% CI		Significance
Intercept	5.06	4.88	5.24	***	0.283	−0.004	0.055	***
Gestational age (weeks)	−0.13	−0.14	−0.11	***	0.259	0.085	0.086	***
Gestational age^2^	0.017	0.016	0.02	***	–	–		
Gestational age^3^	−0.0001	−0.0002	−0.0001	***	–	–		
Mothers’ age (years)	−0.01	−0.02	−0.01	***	−0.003	−0.003	−0.002	***
BMI, kg/m^2^	−0.11	−0.12	−0.11	***	−0.005	−0.021	−0.019	***
Ethnicity								
Caucasian	Reference							
Polynesian	0.21	0.02	0.41	*	−0.034	0.002	0.066	
Mainland Asian	−1.08	−1.16	−1	***	−0.079	−0.143	−0.122	***
Maritime Asian	−0.81	−0.93	−0.7	***	−0.054	−0.116	−0.085	**
Chinese Asian	−0.73	−0.83	−0.62	***	−0.036	−0.102	−0.075	*
Southeast Asian	−0.85	−0.91	−0.79	***	−0.065	−0.12	−0.103	***
African	−1.52	−1.65	−1.39	***	−0.102	−0.246	−0.204	***
Indigenous/Torres Strait Island	−0.51	−0.8	−0.21	***	−0.081	−0.158	−0.049	*
South America	−0.36	−0.66	−0.05	*	0.013	−0.086	0.004	
Southeast Europe	−0.66	−0.74	−0.58	***	−0.031	−0.091	−0.069	*
Eastern Europe	0.43	0.3	0.57	***	0.043	0.028	0.064	
North Africa and the Middle East	−0.17	−0.33	−0.01	*	0.004	−0.04	0.004	
Other	−0.13	−0.4	0.13		−0.047	−0.07	0.004	
Parity								
0	Reference							
1–3	−0.68	−0.73	−0.63	***	−0.044	−0.095	−0.082	***
>3	−1.53	−1.67	−1.39	*	−0.105	−0.273	−0.222	***
Goodness-of-fit statistics								
AIC	549 420					479 939		
SBC	549 621					481 471		
R^2^	0.56					0.70‡		
Summary of the quantile residuals								
Mean	−1.77E-14					−0.004		
Variance	1					0.99		
Coefficient of skewness	0.5					0.045		
Coefficient of kurtosis	4.3					2.8		
Filliben correlation coefficient	0.998					0.9996		

Residual plots of these four models are in online supplemental figure S1Figure S1.

The coefficients for variance (σ), skewness and kurtosis (ν, ‡) are not included.

* Significance codes: 0.1; *0.01≤p<0.05; **0.005≤p<0.01; ***0.001≤p<0.005.

† The linear coefficients or the 95% CI of the smoothing terms should not be interpreted as additive terms exist in the formula.

‡ Generalised R-squared of Nagelkerke (1991).

AICAkaike information criteriaBMIbody mass indexGAMLSSgeneral additive model for location, scale and shapeSBCSchwarz/Bayesian information criteria

### Aim 3: utility of GWG trajectory and centile graphs

**Figure 3:**
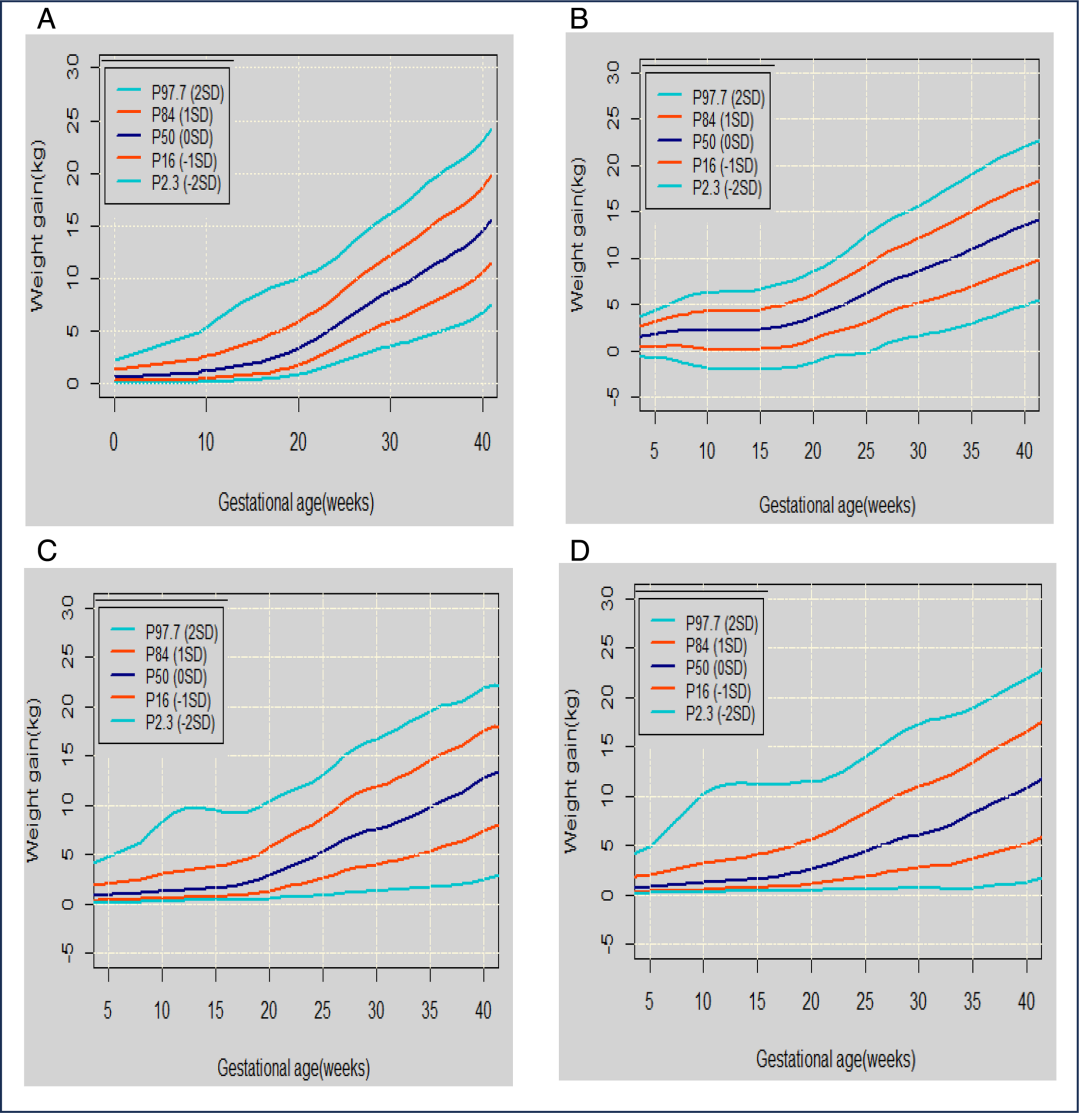
Selected percentiles of weight gain for gestational age for maternal pre-pregnancy underweight (A), normal-weight (B), overweight (C) and obese (D) for Caucasian women.

We produced visual outputs of the GWG trajectories as centile charts determined by the optimal model using the data from the entire dataset (figure 2) and for the Caucasian subgroup by the BMI group (figure 3).

### Sensitivity analysis

To confirm aim 1 result, 14% (5713/39846) of the original dataset with weights measured at the first antenatal visit were analysed on a sensitivity analysis, where these weight measurements become the new baseline. Overlayed on the trajectory centile charts (solid lines; figure 2) are centile charts determined by the sensitivity analysis dataset (dotted lines; figure 2). Before 20 weeks, comparisons were difficult to make due to a high proportion of measurements in the sensitivity analysis now becoming the new baseline (ie, 76% of first-visit weight measurements were in the 1–20-week period). However, beyond 25 weeks gestation, the trajectories from the two models converge, which indicates that the baseline for measuring GWG (pre-pregnancy or first antenatal visit) had little effect on the gestation trajectories in the latter pregnancy stages.

## Discussion

Using a large dataset of routinely collected antenatal data across multiple hospitals serving a diverse ethnic population, we found a very strong correlation between self-reported pre-pregnancy weight and direct weight measurement at the first antenatal visit, providing good evidence of the reliability of pre-pregnancy weight data to underpin our GWG model. We then examined multiple statistical methods and determined the optimal model (GAMLSS) to generate accurate GWG trajectories for this diverse population. This statistical method is suitable for modelling outcomes that are nonlinear and possess distributional complexities and allows for the inclusion of key maternal factors that are associated with GWG. The algorithm was then applied to demonstrate the feasibility of producing GWG centile charts for any given combination of factors (ie, age, BMI and parity), and we demonstrate how to do this using our large study population, and also separately for one subgroup (Caucasians).

The high intraclass correlation between self-reported weight at the start of the pregnancy and weight at first measurement (between 4 and 10 weeks in our dataset) revealed that using self-reported weight in modelling GWG is valid, especially due to the large size of our data. Furthermore, replacing self-reported weight with the first measured weight resulted in many initial values being made redundant, which could make the predictions of centile trajectories less reliable.

GWG has been shown to differ substantially in women according to age and ethnicity.^6^,^820 21^ Whether these differences are caused by biological differences in the process of nutrient absorption or fat deposition or due to socioeconomic, cultural and nutritional factors is unclear.^22^ Our analysis and others consistently show that parity is also associated with GWG as the relationship between parity and GWG is associated with BMI. Therefore, there is strong evidence to include these important factors within trajectory modelling for GWG as we did herein. Prior research on GWG modelling includes a study on 218 216 underweight, normal-weight, overweight and obese women using data from cohorts from Europe, North America and Oceania^13^ which also used the GAMLSS method to predict GWG. Here, we extend prior work by exploring and determining the optimal statistical methods that accommodate the complexities in GWG data, assessing the importance of including covariates in the model. Several other studies have analysed the trajectory of GWG including a hospital-based study that developed GWG charts for 4246 overweight and obese US women.^23^ The INTERGROWTH-21st Project evaluated GWG among 3097 normal-weight women from Brazil, China, India, Italy, Kenya, Oman, the UK and the USA.^22^ Another study analysed a large population-based cohort of underweight, normal-weight, overweight and obese women of 141 767 Swedish women with term, non-anomalous, singleton pregnancies and no pre-existing hypertension or diabetes.^24^ However, these existing studies either used methods without accounting for violations in distributional assumptions or ignored the contribution of key maternal covariates to GWG. It was also unclear which modelling methods perform best due to the lack of evidence on model comparisons.

Here, we have made advances in the field by identifying the GAMLSS method as optimal in managing the complexities of GWG data. Furthermore, we assess the contribution made by covariates that are associated with GWG in modelling GWG trajectories. Nonlinear GWG trajectory modelling has been reported previously; however, the approaches applied have significant limitations.^25^ Piece-wise linear regression^26^ requires choosing arbitrary gestational age cut points, assuming the rate of GWG is linear within each cut point-defined interval and estimating abrupt changes at these cut points, which may be biologically improbable. Other more complex approaches, such as latent class models,^27^ may better describe growth trajectories, but the interpretation of parameters is imprecise, and the utility of such models is limited. Nonlinear mixed-effects models such as latent model structures have limited flexibility to cater to the intricate nature of GWG data. The Super-Imposition by Translation and Rotation growth model has also been used to analyse GWG trajectories of 3470 normal-weight, overweight and obese women,^28^ but is too restrictive and is applicable when only one explanatory variable is available. Overall, we propose that the GAMLSS model is optimal for managing complex GWG data, especially as the residual plots for the other models showed non-normality and skewness (online supplemental figure S1). The main characteristic of GAMLSS models is the ability to allow the location, scale and shape of the distribution of the response variable to vary according to the values of explanatory variables. Additionally, it is semiparametric, which offers several advantages including that it can use a wider range of distributions, including the distribution of the exponential family and other distributions, and in fact, our two best models were GAMLSS: one fitted with penalised B-splines and the other with cubic splines.

Using the optimal model, we demonstrate how to generate centile charts for the sample population. While our sample size was large and observed values can also produce centile graphs, the key advantage of using model-based values is their ability to create more personalised and representative centiles tailored to an individual’s characteristics, as model input factors are maternal age, pre-pregnancy BMI, parity and ethnicity (country of birth). Additionally, model-based values enable the creation of centile charts even when data points are limited, thus offering the potential for more personalised charts that can be applied to a broader range of individuals. We have also extended prior work to demonstrate the feasibility of using the model to produce individualised percentile charts depending on the women’s characteristics. This model can produce up to 15 000 individualised charts. This capability of the model will be used moving forward through incorporation into a computerised tool, whereby an individual woman and their health professional can input individual characteristics, and an accurate percentile chart can be generated—a strong advance towards prospective healthy GWG management and to personalised care supported by data-driven methods.

The next steps in this research programme include enhancing this GWG trajectory model dynamically: it will be validated and calibrated annually using new birthing data (approximately 12 000 births/annum at Monash Health), which will continuously improve model accuracy and can accommodate potential policy and demographic changes that may take place in the future. Also, lifestyle interventions targeting recommended GWG by BMI category are known to improve health outcomes and are cost-effective. Future work is currently underway developing a tool incorporating the optimal algorithm developed in this study to assist with providing timely interventions by antenatal care providers to reduce the risks of adverse maternal and neonatal effects due to inadequate or excessive GWG. We hope to improve the accuracy of determining ethnicity by exploring self-identified ethnicity in the model.

Setting and reaching GWG targets requires a model that can accurately show the expected GWG trajectory for each woman and also display the NAM-recommended range at any stage of the pregnancy to guide the healthy GWG recommended for each woman throughout her pregnancy. It is anticipated that such a tool will be valuable alongside the implementation of healthy lifestyle interventions in pregnancy to improve the GWG targets and health of women and the next generation.

### Strengths and limitations

The strengths of this study are the description of the pattern of weight gain throughout pregnancy in a large sample of pregnant women from diverse ethnic backgrounds. This is a general population-based cohort study; therefore, we had a good representation across all socioeconomic groups; hence, we had a fair representation of the prevalence of inadequate and excessive GWG. We examined an extensive array of analytical methods to derive the best model for predicting GWG. However, we had limited data on weight measurements at the start of the pregnancy available for this analysis (online supplemental table S1). Clinical measurements of height and weight are considered the gold standard that produces more accurate data on pre-pregnancy BMI than self-reported measurements. However, self-reported weight and height have been determined to be a reasonable proxy,^29^ especially for a reasonably large sample size. Furthermore, the correlation between self-reported weight at the start of pregnancy and weight at first measurement was consistent using the intraclass correlation coefficient. As this is routine health data, we were unable to examine if women change their weight-related behaviours during pregnancy influenced by the attending physicians and others; however, we aim to examine this prospectively in a pragmatic trial using this model to predict GWG.

## Conclusion

As data on GWG become more accessible, it is crucial for researchers and healthcare providers to have clear, evidence-based methods for analysing this data and identifying those at risk of suboptimal GWG during pregnancy. This research lays the groundwork for future studies by developing algorithms that can handle the complexities of GWG, including factors like ethnicity and parity. These algorithms will enable more accurate, personalised estimates of GWG trajectories, ultimately improving the health of both women and their babies. We are currently developing a GWG calculator that will be available for use by healthcare professionals and patients alike.

## supplementary material

10.1136/bmjopen-2024-088664 online supplemental file 1

## Data Availability

Data may be obtained from a third party and are not publicly available.
