# Supplementary material for: Comparison of statistical modelling methods for population-level gestational weight gain trajectories in ethnically diverse women in southeast Melbourne, Australia
Source: BMJ Open. 2025 Mar 13;15(3):e088664. doi: 10.1136/bmjopen-2024-088664 (PMC11906984; doi:10.1136/bmjopen-2024-088664)
Supplement: online supplemental file 1 [file bmjopen-15-3-s001.docx]

**Supplemental Material**

**Table of contents**

Number of women by visit and gestational age (Table S1) ………………………………………. ………………………2

Residual plots for selected models. (Figure S1).…………………………………………………………………………….3

Model indices for the neural network model (Table S2, Figure S2, S3) ………………………………………………….4

Model indices for the fractional polynomial model (Table S3, Figure S4) ……….……………………………………….5

Model indices for the generalised additive model (Table S4, Figure S5).....................................................................6

The plot of the fitted terms for model Box Cox t-original distribution (Figure S2) ........................................................7

Countries by region (number and percentage) (Table S5) …………….………………….…………………………….….8-9

Supplementary Methods for the GAMLSS model……………..………………………………………………….………...10-11

GAMLASS: Analysis of distributions (Table S6) ……………….………………….……………………………….………..12

RECORD STATEMENT CHECKLIST (Table S7) ……………….………………….……………………………….………14

**Abbreviations**

GWG - Gestational Weight Gain

BMI - Body Mass Index

GAM - Generalised Additive Models

GAMLSS - Generalised Additive Models for Location Scale and Shape

|  | | | | | | | | | | | | | | | | | | | | | | |
| --- | --- | --- | --- | --- | --- | --- | --- | --- | --- | --- | --- | --- | --- | --- | --- | --- | --- | --- | --- | --- | --- | --- |
|  | **No. of visits** | | | | | | | | | | | | | | | | | | | | | |
| Gest. week | **1** | **2** | **3** | **4** | **5** | **6** | **7** | **8** | **9** | **10** | **11** | **12** | **13** | **14** | **15** | **16** | **17** | **18** | **19** | **20** | **21** | Total |
| 1-9 | 31 | 52 | 6 | 0 | 0 | 0 | 0 | 0 | 0 | 0 | 0 | 0 | 0 | 0 | 0 | 0 | 0 | 0 | 0 | 0 | 0 | 89 |
| 10 | 91 | 231 | 16 | 1 | 0 | 0 | 0 | 0 | 0 | 0 | 0 | 0 | 0 | 0 | 0 | 0 | 0 | 0 | 0 | 0 | 0 | 339 |
| 11 | 117 | 410 | 29 | 5 | 3 | 2 | 1 | 1 | 1 | 0 | 0 | 0 | 0 | 0 | 0 | 0 | 0 | 0 | 0 | 0 | 0 | 569 |
| 12 | 135 | 540 | 35 | 1 | 1 | 0 | 0 | 0 | 0 | 0 | 0 | 0 | 0 | 0 | 0 | 0 | 0 | 0 | 0 | 0 | 0 | 712 |
| 13 | 146 | 652 | 54 | 9 | 6 | 0 | 0 | 0 | 0 | 0 | 0 | 0 | 0 | 0 | 0 | 0 | 0 | 0 | 0 | 0 | 0 | 867 |
| 14 | 172 | 761 | 81 | 8 | 3 | 0 | 0 | 0 | 0 | 0 | 0 | 0 | 0 | 0 | 0 | 0 | 0 | 0 | 0 | 0 | 0 | 1,025 |
| 15 | 172 | 783 | 147 | 8 | 2 | 0 | 1 | 0 | 0 | 0 | 0 | 0 | 0 | 0 | 0 | 0 | 0 | 0 | 0 | 0 | 0 | 1,113 |
| 16 | 217 | 969 | 191 | 9 | 4 | 1 | 1 | 0 | 0 | 0 | 0 | 0 | 0 | 0 | 0 | 0 | 0 | 0 | 0 | 0 | 0 | 1,392 |
| 17 | 152 | 693 | 155 | 5 | 3 | 0 | 0 | 0 | 0 | 0 | 0 | 0 | 0 | 0 | 0 | 0 | 0 | 0 | 0 | 0 | 0 | 1,008 |
| 18 | 168 | 622 | 117 | 12 | 2 | 1 | 0 | 0 | 0 | 0 | 0 | 0 | 0 | 0 | 0 | 0 | 0 | 0 | 0 | 0 | 0 | 922 |
| 19 | 130 | 527 | 130 | 14 | 6 | 2 | 2 | 0 | 0 | 0 | 0 | 0 | 0 | 0 | 0 | 0 | 0 | 0 | 0 | 0 | 0 | 811 |
| 20 | 136 | 845 | 244 | 38 | 7 | 1 | 1 | 1 | 1 | 0 | 0 | 0 | 0 | 0 | 0 | 0 | 0 | 0 | 0 | 0 | 0 | 1,274 |
| 21 | 269 | 2,111 | 885 | 139 | 30 | 7 | 2 | 0 | 0 | 0 | 0 | 0 | 0 | 0 | 0 | 0 | 0 | 0 | 0 | 0 | 0 | 3,443 |
| 22 | 337 | 2,906 | 1,506 | 329 | 63 | 9 | 6 | 1 | 0 | 0 | 0 | 0 | 0 | 0 | 0 | 0 | 0 | 0 | 0 | 0 | 0 | 5,157 |
| 23 | 107 | 708 | 383 | 62 | 10 | 2 | 3 | 1 | 1 | 1 | 1 | 0 | 0 | 0 | 0 | 0 | 0 | 0 | 0 | 0 | 0 | 1,279 |
| 24 | 98 | 298 | 178 | 43 | 9 | 4 | 4 | 0 | 0 | 0 | 0 | 0 | 0 | 0 | 0 | 0 | 0 | 0 | 0 | 0 | 0 | 634 |
| 25 | 56 | 213 | 136 | 56 | 15 | 8 | 3 | 1 | 1 | 0 | 0 | 0 | 0 | 0 | 0 | 0 | 0 | 0 | 0 | 0 | 0 | 489 |
| 26 | 92 | 306 | 363 | 111 | 22 | 4 | 3 | 1 | 0 | 0 | 0 | 0 | 0 | 0 | 0 | 0 | 0 | 0 | 0 | 0 | 0 | 902 |
| 27 | 243 | 1,261 | 1,538 | 591 | 132 | 27 | 12 | 0 | 0 | 1 | 1 | 1 | 1 | 0 | 0 | 0 | 0 | 0 | 0 | 0 | 0 | 3,808 |
| 28 | 488 | 2,382 | 3,116 | 1,405 | 349 | 53 | 36 | 11 | 11 | 2 | 2 | 0 | 0 | 0 | 0 | 0 | 0 | 0 | 0 | 0 | 0 | 7,855 |
| 29 | 112 | 394 | 532 | 288 | 88 | 24 | 10 | 5 | 3 | 2 | 0 | 1 | 1 | 1 | 0 | 0 | 0 | 0 | 0 | 0 | 0 | 1,461 |
| 30 | 185 | 435 | 957 | 811 | 347 | 74 | 28 | 7 | 5 | 0 | 0 | 0 | 0 | 0 | 0 | 0 | 0 | 0 | 0 | 0 | 0 | 2,849 |
| 31 | 350 | 827 | 2,053 | 2,060 | 904 | 220 | 70 | 19 | 14 | 4 | 4 | 1 | 1 | 0 | 1 | 0 | 0 | 0 | 0 | 0 | 0 | 6,528 |
| 32 | 379 | 554 | 1,356 | 1,325 | 760 | 255 | 61 | 16 | 11 | 4 | 3 | 3 | 2 | 1 | 1 | 0 | 0 | 0 | 0 | 0 | 0 | 4,731 |
| 33 | 556 | 378 | 1,256 | 1,552 | 1,307 | 578 | 176 | 48 | 38 | 8 | 7 | 2 | 2 | 0 | 0 | 0 | 0 | 0 | 0 | 0 | 0 | 5,908 |
| 34 | 684 | 408 | 1,610 | 1,876 | 1,730 | 900 | 269 | 76 | 49 | 14 | 13 | 3 | 3 | 1 | 1 | 2 | 2 | 0 | 0 | 0 | 0 | 7,641 |
| 35 | 726 | 242 | 1,125 | 1,129 | 1,150 | 939 | 397 | 130 | 41 | 24 | 23 | 2 | 2 | 2 | 2 | 0 | 0 | 1 | 1 | 0 | 0 | 5,936 |
| 36 | 764 | 215 | 1,233 | 1,312 | 1,454 | 1,404 | 706 | 225 | 70 | 32 | 30 | 14 | 12 | 1 | 1 | 0 | 0 | 0 | 0 | 1 | 1 | 7,475 |
| 37 | 351 | 99 | 559 | 746 | 934 | 926 | 700 | 317 | 114 | 25 | 13 | 12 | 12 | 8 | 8 | 1 | 1 | 0 | 0 | 0 | 0 | 4,826 |
| 38 | 306 | 51 | 500 | 751 | 882 | 1,013 | 1,023 | 601 | 183 | 68 | 26 | 15 | 17 | 2 | 2 | 3 | 3 | 0 | 0 | 0 | 0 | 5,446 |
| 39 | 149 | 16 | 272 | 394 | 433 | 518 | 514 | 493 | 271 | 78 | 25 | 3 | 3 | 5 | 5 | 0 | 0 | 1 | 1 | 0 | 0 | 3,181 |
| 40-43 | 64 | 2 | 128 | 171 | 220 | 215 | 268 | 231 | 174 | 104 | 25 | 11 | 4 | 2 | 2 | 0 | 0 | 0 | 0 | 0 | 0 | 1,621 |
| Total | 7983 | 20891 | 20891 | 15261 | 10876 | 7187 | 4297 | 2185 | 988 | 367 | 173 | 68 | 60 | 23 | 23 | 6 | 6 | 2 | 2 | 1 | 1 | 91291 |

Table S1. Number of women by visit and gestational age

Figure S1: Residual plots for the 4 selected models displayed in Table 2.

**C. Cubic polynomial**

**D. GAMLSS Box-Cox t-original penalised splines**

1. **A. Linear regression**


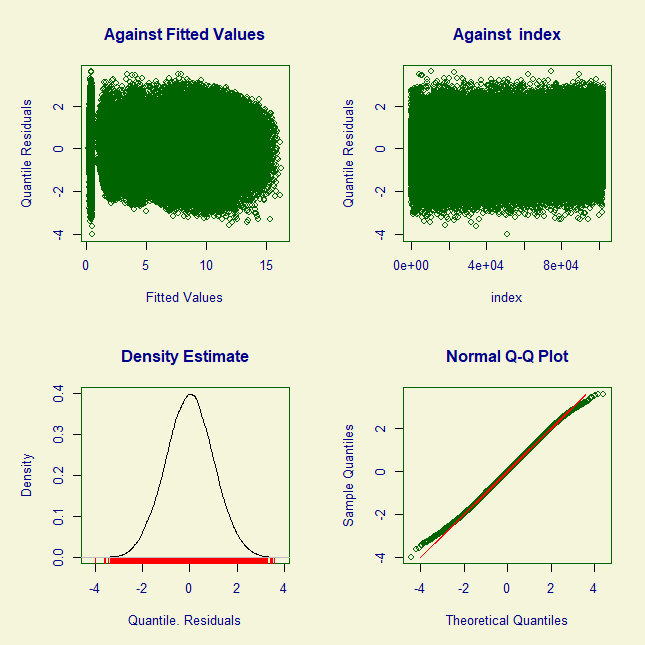


**B. GAMLSS Box-Cox t-original cubic spline**


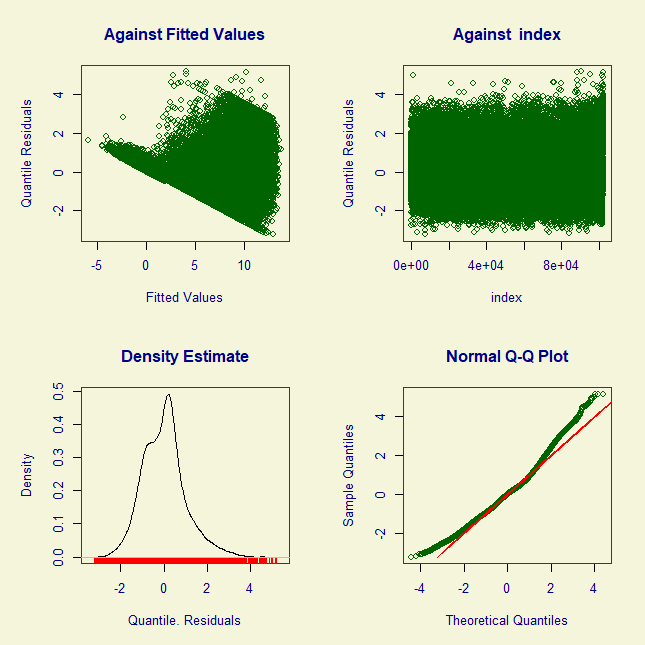

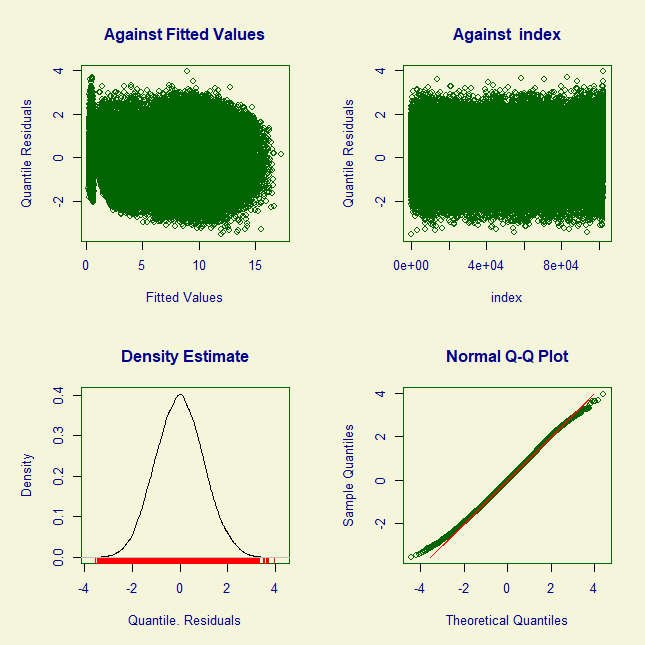

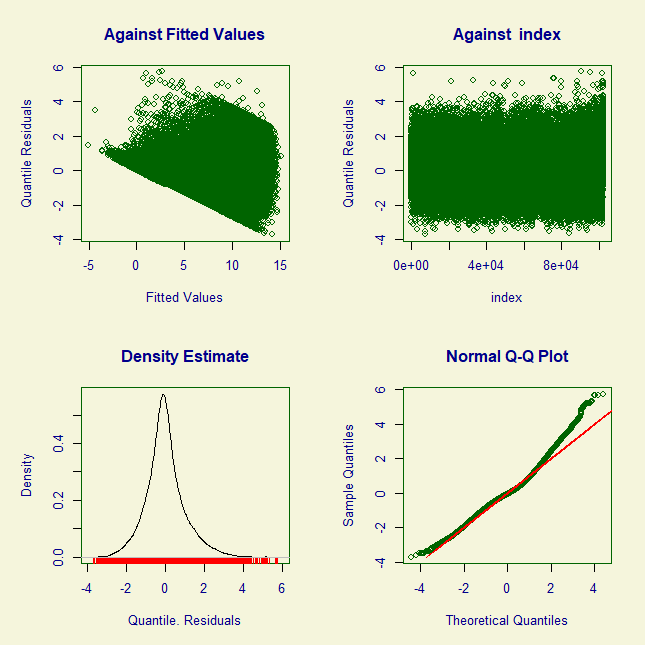


Figure S2 Neural network


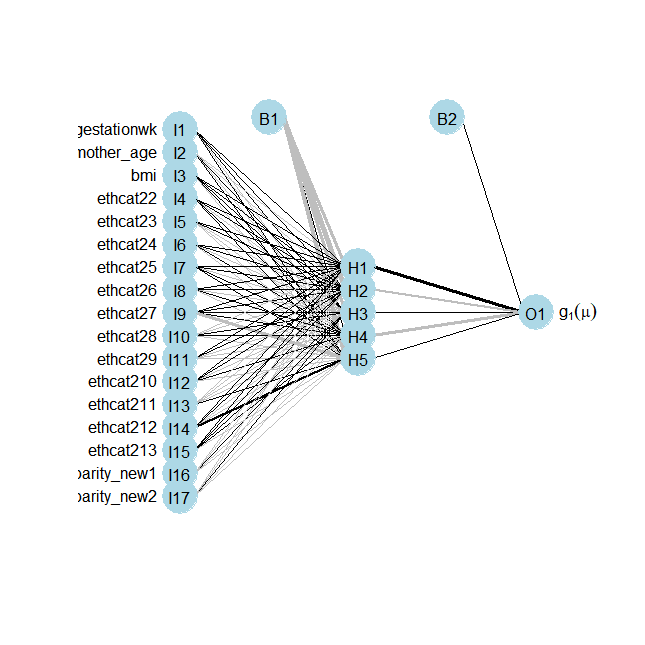


Gestational wks.

Mothers age

BMI

Polynesian

Mainland Asian

Maritime Asian

NE Asian

SC. Asian

African

Indigenous/Torres St.

South America

SE Europe

Eastern Europe

North Arica and ME

Other

Parity 1-3

Parity >=4

The lines connecting the neurons are the weights between them where black lines represent positive weights and grey lines represent negative weights. The thickness of the lines specifies the strength the weight: thick lines have a greater absolute value than thin lines.

Figure S3. Residual plots for Neural network model.


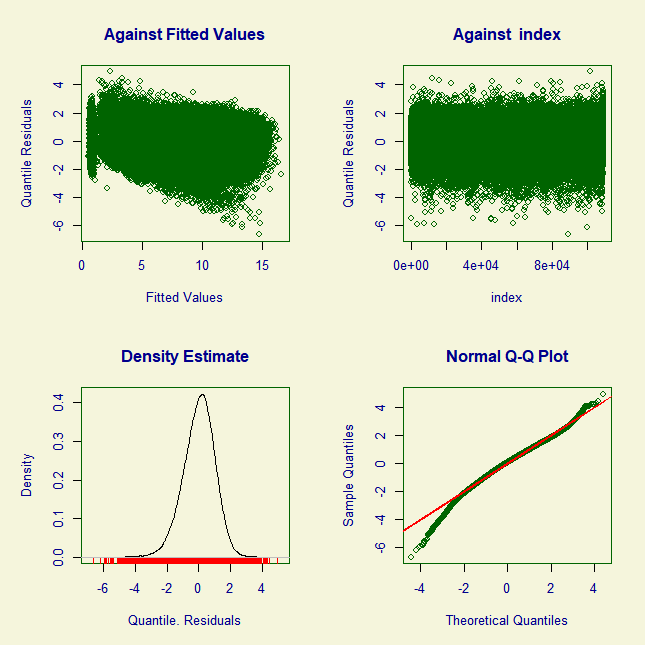
 Table S2. Model indices for Neural network model

| AIC | 510809 |
| --- | --- |
| SBC | 512672 |
| R^2^ | 71.5 |
| Mean | >0.001 |
| variance | 1.0 |
| Coef.of skewness | -0.37 |
| Coef. Of kurtosis | 3.7 |
| Filliben correlation coefficient | 0.996 |
| AIC | 510809 |
| SBC | 512672 |
| R^2^ | 71.5 |
| Mean | >0.001 |
| variance | 1.0 |
| Coef.of skewness | -0.37 |
| Coef. Of kurtosis | 3.7 |
| Filliben correlation coefficient | 0.996 |

| AIC | 510809 |
| --- | --- |
| SBC | 512672 |
| R^2^ | 71.5 |
| Mean | >0.001 |
| variance | 1.0 |
| Coef.of skewness | -0.37 |
| Coef. Of kurtosis | 3.7 |
| Filliben correlation coefficient | 0.996 |

Table S3. Model indices for fractional polynomial model

| AIC | 518695 |
| --- | --- |
| SBC | 517548 |
| R^2^ | 0.67 |
| Mean | -0.014 |
| variance | 0.97 |
| Coef.of skewness | 0.1 |
| Coef. Of kurtosis | 2.9 |
| Filliben correlation coefficient | 0.999 |

Figure S4. Residual plots for the fractional polynomial model (best of three).


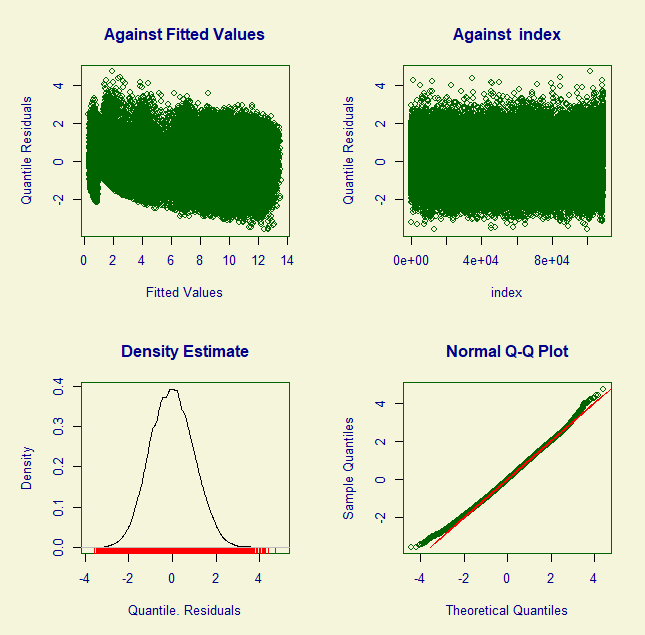


Table S4. Model indices for generalised additive model (GAM)

| AIC | 588237 |  |  |  |
| --- | --- | --- | --- | --- |
| BIC | 588705 |  |  |  |
| R^2^ | 0.56 |  |  |  |
| Smoothed parameters* | k' | edf | k-index | p-value |
| s(gestationwk) | 19 | 11.91 | 1 | 0.52 |
| s(mother_age) | 19 | 10.51 | 1.02 | 0.92 |
| s(bmi) | 19 | 8.53 | 1 | 0.61 |

*Family: Gaussian, Basis functions(k) = 20, Method: REML) & model diagnostics. (k’= maximum no. of basis functions, edf = estimated no. of basis functions, k-index ≈1 & p-val. > 0.05 = good fit

Figure S5. Residual plots for the generalised additive model (GAM) model.


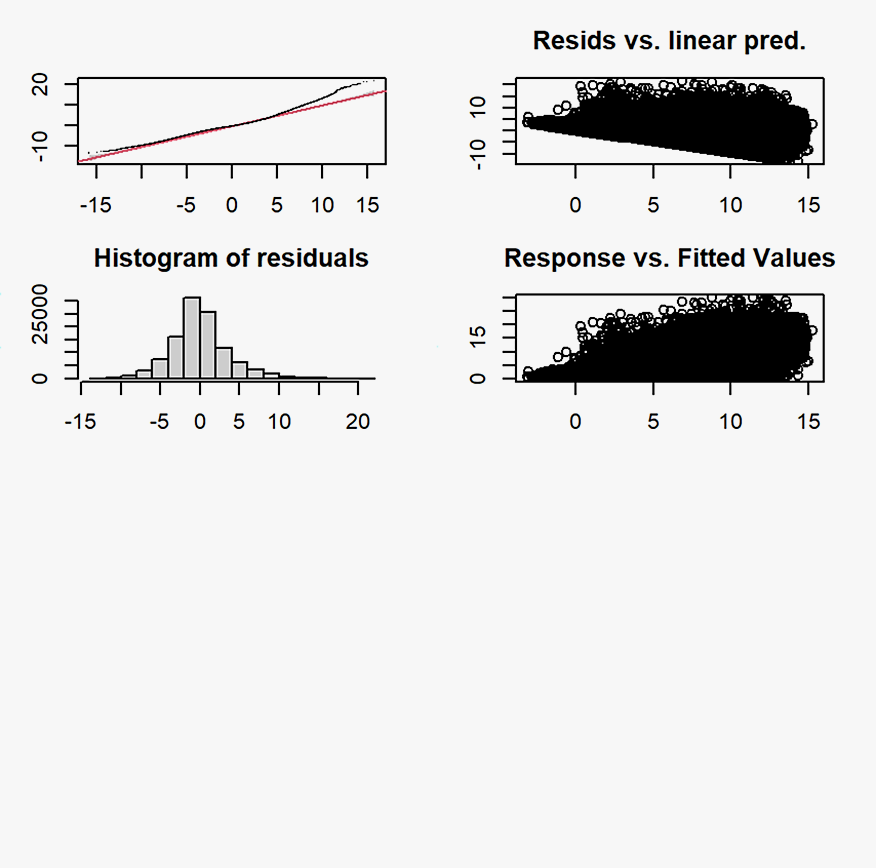


Figure S6: A plot of the fitted terms for model Box Cox t-original distribution


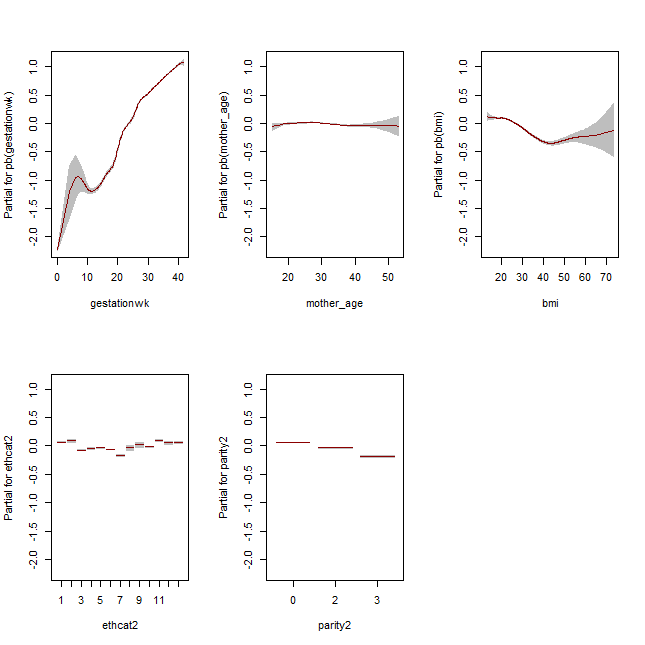


Ethnicity: 1 = Caucasian, 2 = Polynesian, 3 = Mainland Asian, 4 = Maritime Asian, 5 = Chinese, 6 = Southeast Asian, 7 = African, 8 = indigenous (Australian), 9 = South Central American, 10 = Central Asian, 11= Southeastern Europe, 12 = North Africa/Mideast, 13 = Other


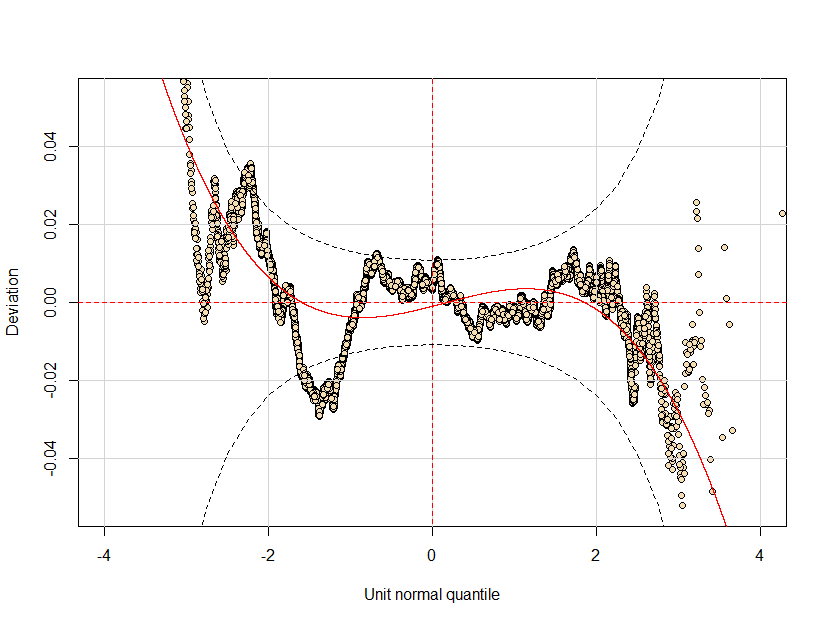
Figure S7: A plot of the fitted terms for model Box Cox t-original distribution

Table S5 Countries by region (number and percentage)

| **Caucasian** | **Freq.** | **Percent** |  | **Polynesian/Islander** | **Freq.** | **Percent** |
| --- | --- | --- | --- | --- | --- | --- |
| Australia | 13,044 | 86.43 |  | Cook Islands | 143 | 26.09 |
| Australia (inc. External Territories) | 189 | 1.23 |  | Fiji | 169 | 30.84 |
| Austria | 3 | 0.02 |  | Nauru | 2 | 0.36 |
| Belgium | 3 | 0.02 |  | Papua New Guinea | 6 | 1.09 |
| Canada | 47 | 0.31 |  | Samoa | 203 | 37.04 |
| Denmark | 6 | 0.04 |  | Samoa, American | 8 | 1.46 |
| England | 239 | 1.58 |  | Solomon Islands | 2 | 0.36 |
| Finland | 4 | 0.03 |  | Tonga | 13 | 2.37 |
| France | 15 | 0.1 |  | Vanuatu | 2 | 0.36 |
| Germany | 71 | 0.47 |  |  |  |  |
| Iceland | 1 | 0.01 |  | **Mainland South-East Asian** |  |  |
| Ireland | 71 | 0.47 |  | Cambodia | 953 | 33.65 |
| Italy | 30 | 0.2 |  | Laos | 10 | 0.35 |
| Luxembourg | 1 | 0.01 |  | Myanmar | 258 | 9.11 |
| Netherlands | 20 | 0.13 |  | Thailand | 286 | 10.1 |
| New Caledonia | 1 | 0.01 |  | Vietnam | 1,325 | 46.79 |
| New Zealand | 1,098 | 7.28 |  |  |  |  |
| Northern Ireland | 7 | 0.05 |  | **Maritime SE Asian** |  |  |
| Norway | 2 | 0.01 |  | Indonesia | 241 | 16.02 |
| Portugal | 11 | 0.07 |  | Malaysia | 488 | 32.45 |
| Scotland | 34 | 0.23 |  | Philippines | 700 | 46.54 |
| Sweden | 10 | 0.07 |  | Singapore | 75 | 4.99 |
| Switzerland | 5 | 0.03 |  |  |  |  |
| United Kingdom, Channel Islands & Isle | 78 | 0.52 |  | **NE Asian** |  |  |
| United States of America | 92 | 0.61 |  | China (Excludes SARS & Taiwan) | 1,402 | 74.22 |
| Wales | 10 | 0.07 |  | Chinese Asia (includes Mongolia) | 14 | 0.74 |
| **Southern and central Asia** |  |  |  | Hong Kong | 112 | 5.93 |
| Bangladesh | 182 | 2.16 |  | Japan | 90 | 4.76 |
| India | 5,494 | 65.22 |  | N. Korea | 29 | 1.54 |
| Maldives | 4 | 0.05 |  | S. Korea | 165 | 8.73 |
| Nepal | 179 | 2.12 |  | Macau | 4 | 0.21 |
| Pakistan | 1,025 | 12.17 |  | Mongolia | 1 | 0.05 |
| Sri Lanka | 1,540 | 18.28 |  | Taiwan | 72 | 3.81 |
|  |  |  |  |  |  |  |
| **Southeast Europe** |  |  |  | **Africa** |  |  |
| Albania | 84 | 11.57 |  | Angola | 1 | 0.08 |
| Lithuania | 4 | 0.53 |  | Botswana | 1 | 0.08 |
| Bosnia-Herzegovina | 216 | 29.75 |  | Burundi | 10 | 0.78 |
| Bulgaria | 3 | 0.41 |  |  |  |  |
| Croatia | 59 | 8.13 |  | Cameroon | 3 | 0.23 |
| Yugoslav | 90 | 12.4 |  | Congo | 9 | 0.55 |
| Greece | 55 | 7.58 |  | Cote d'Ivoire | 4 | 0.31 |
|  |  |  |  | Djibouti | 1 | 0.08 |
| Moldova | 6 | 0.83 |  | Eritrea | 22 | 1.72 |
| Montenegro | 2 | 0.28 |  | Ethiopia | 189 | 14.79 |
| Romania | 117 | 16.12 |  | Ghana | 9 | 0.7 |
| Serbia | 82 | 11.29 |  | Guinea | 3 | 0.23 |
| Slovenia | 7 | 0.96 |  | Guyana | 1 | 0.08 |
| Czech Republic | 4 | 0.53 |  | Kenya | 66 | 5.16 |
| **South/Central America** |  |  |  | Liberia | 34 | 2.67 |
| Argentina | 19 | 9.27 |  | Malawi | 4 | 0.31 |
| Brazil | 14 | 6.83 |  | Mauritius | 185 | 14.46 |
| Chile | 44 | 21.46 |  | Mozambique | 1 | 0.08 |
| Colombia | 50 | 24.39 |  | Namibia | 2 | 0.16 |
| El Salvador | 28 | 13.66 |  | Niger | 1 | 0.08 |
| Mexico | 16 | 7.8 |  | Nigeria | 45 | 3.52 |
| Paraguay | 2 | 0.98 |  | Rwanda | 6 | 0.47 |
| Peru | 16 | 7.8 |  | Seychelles | 9 | 0.7 |
| Uruguay | 6 | 2.93 |  | Sierra Leone | 17 | 1.33 |
| Venezuela | 10 | 4.88 |  | Somalia | 54 | 4.24 |
| Honduras | 3 | 0.41 |  | South Sudan | 41 | 3.21 |
| **Central Asia** |  |  |  | Sudan | 431 | 33.7 |
| Afghanistan | 2,875 | 96.8 |  | Uganda | 13 | 1.02 |
| Armenia | 12 | 0.4 |  | Yemen | 9 | 0.7 |
| Azerbaijan | 2 | 0.07 |  | Zambia | 10 | 0.79 |
| Georgia | 1 | 0.03 |  | Zimbabwe | 98 | 7.66 |
| Kazakhstan | 8 | 0.27 |  |  |  |  |
| Kyrgyzstan | 5 | 0.17 |  | **N. African & Mid. East** |  |  |
| Tajikistan | 1 | 0.03 |  | Algeria | 2 | 0.28 |
| Turkey | 60 | 2.02 |  | Bahrain | 6 | 0.8 |
| Uzbekistan | 6 | 0.2 |  | Egypt | 111 | 14.8 |
|  |  |  |  | Iran | 245 | 32.67 |
|  |  |  |  | Iraq | 122 | 16.27 |
|  |  |  |  | Jordan | 15 | 2 |
|  |  |  |  | Kuwait | 21 | 2.8 |
|  |  |  |  | Lebanon | 94 | 12.53 |
|  |  |  |  | Libya | 2 | 0.27 |
|  |  |  |  | Morocco | 8 | 1.07 |
|  |  |  |  | Oman | 6 | 0.8 |
|  |  |  |  | Qatar | 3 | 0.4 |
|  |  |  |  | Saudi Arabia | 32 | 4.27 |
|  |  |  |  | Syria | 34 | 4.53 |
|  |  |  |  | Tunisia | 2 | 0.27 |
|  |  |  |  | United Arab Emirates | 41 | 5.47 |

Supplementary Methods

A GAMLSS model is a general regression model which assumes that the response (dependent) variable has any parametric distribution. In addition, all the parameters of the distribution of the response variable can be modelled as functions of the available explanatory variables. This is in contrast to GLM’s and GAM’s where the distribution of the response variable is restricted to the exponential family of distributions and only the mean (a location parameter) of the distributions is modelled. Therefore, the main characteristic of GAMLSS models is the ability to allow the location, scale and shape of the distribution of the response variable to vary according to the values of explanatory variables. and it is semiparametric, which offers several advantages. Firstly, it can use a wider range of distributions, including the distribution of the exponential family and other distributions. Secondly, all the parameters (𝜇, 𝜎, 𝑣, 𝜏) can be modelled based on the distribution used (formulas a-d). The model analysed in the study used gestational age, BMI, mother's age, parity, and region/COB as explanatory variables, with GWG as the response variable. I chose the four-parameter Box Cox t-distribution because the response variable GWG exhibited varying kurtosis, so a four-parameter distribution [BCT ($\mu,\sigma, \nu, \tau$)] was deemed most appropriate for the BMI categories underweight, overweight and obese. However, for the BMI category normal weight normal distribution was deemed appropriate as other distributions failed to converge. The function NO() defines the normal distribution, a two parameter distribution, for a gamlss. Family object to be used in GAMLSS fitting using the function gamlss (), with mean equal to the parameter mu and sigma equal the standard deviation. This decision was justified as it produced the optimal goodness of fit indices. This analysis employed the P-spline smoother (pb), which utilises local maximum likelihood estimation to estimate smoothing parameters and is considered the most reliable method.

Rigby and Stasinopoulos (2005) define an original formulation of a GAMLSS model as follows.

Response variable observations $Y_{1}, Y_{2},...,Y_{n}$ are independent with

$$y ind\sim D(\mu,\sigma, \nu, \tau)$$

For i = 1..., n, where D is any distribution with four parameters for the t-distribution.

For k = 1, 2, 3, 4, let g_k_ (.) be a known monotonic link function relating a distribution parameter to a predictor $\eta_{k}$ where

$g_{1(\mu)}=\eta_{1}= x_{1}\beta_{1}+ \sum_{j=1}^{j1} z_{j1}\gamma_{j1}$ (a)

$g_{2(o)}=\eta_{2}= x_{2}\beta_{2}+ \sum_{j=1}^{j2} z_{j2}\gamma_{j2}$ (b)

$g_{3(\nu)}=\eta_{3}= x_{3}\beta_{3}+ \sum_{j=1}^{j3} z_{j3}\gamma_{j3}$ (c)

$g_{4(\tau)}=\eta_{4}= x_{4}\beta_{4}+ \sum_{j=1}^{j4} z_{j4}\gamma_{j4}$ (d)

where $X_{k}$ is a known design matrix, $\beta_{k}$ = ($\beta_{k1,\ldots\ldots}$ $\beta_{k{j'}_{k}}$)^⊤^ is a parameter vector of length ${{j'}_{k}}^{'} , s_{kj}$ is a smooth non-parametric function of variable $X_{kj}$ and the $X_{kj}$’s are vectors of length n, for k = 1, 2, 3, 4 and j = 1,... $J_{k}.$ That is, a GAMLSS model allows the modelling of the parameters of the distribution as linear, that is, $X_{k}\beta_{k}$ or smooth term functions $S_{kj}(X_{kj})$ for k = 1, 2, 3, 4.

The Box-Cox t-distribution:

$$Y\sim BCT(\mu,\sigma, \nu, \tau)$$

$$\mu=S_{1}(x)$$

$$log(\sigma)=S_{2}(x)$$

$$\nu=S_{3}(x)$$

$$log(\tau)=S_{4}(x)$$

Where s1(.) is a smooth surface and s2(.), s3(.) and s4(.) are smooth functions.

**Table S6. Model performance with GAMLSS distributions.**

| **Distribution** | **Model indices** | | |
| --- | --- | --- | --- |
|  | global deviance | Akaike criteria | Schwartz's Bayesian Criterion |
| BCTo* | 473358.1 | 473685.8 | 475248.6 |
| BCCGo | 912846.3 | 913341.5 | 915702.6 |
| NO | 498866.4 | 499014.9 | 499723 |
| BCPEo | did not converge | did not converge | did not converge |

*Optimal performing distribution was BCTo.

BCTo=Box-Cox t original, BCCGo= Box-Cox Cole-Green orig, NO= normal, BCPEo= Box-Cox Power Exponential. original.

**Table S7. RECORD statement checklist of items, extended from the STROBE statement, reported in observational studies using routine health data.**

|  | **Item No.** | **STROBE items** | **Location in manuscript where items are reported** | **RECORD items** | **Location in manuscript where items are reported** |  |
| --- | --- | --- | --- | --- | --- | --- |
| **Title and abstract** | | | | | | |
|  | 1 | (a) Indicate the study’s design with a commonly used term in the title or the abstract (b) Provide in the abstract an informative and balanced summary of what was done and what was found | Page 1/3 Methods | RECORD 1.1: The type of data used should be specified in the title or abstract. When possible, the name of the databases used should be included.  RECORD 1.2: If applicable, the geographic region and timeframe within which the study took place should be reported in the title or abstract.  RECORD 1.3: If linkage between databases was conducted for the study, this should be clearly stated in the title or abstract. | Page 1/3 Methods  Page 3 Methods  NA |  |
| **Introduction** | | | | | | |
| Background rationale | 2 | Explain the scientific background and rationale for the investigation being reported | Introduction Page 3-4 |  |  |  |
| Objectives | 3 | State specific objectives, including any prespecified hypotheses | Page 3 |  |  |  |
| **Methods** | | | | | | |
| Study Design | 4 | Present key elements of study design early in the paper | Page 1 Design and Setting |  |  |  |
| Setting | 5 | Describe the setting, locations, and relevant dates, including periods of recruitment, exposure, follow-up, and data collection | Page 3 Methods |  |  |  |
| Participants | 6 | *(a) Cohort study* - Give the eligibility criteria, and the sources and methods of selection of participants. Describe methods of follow-up  *Case-control study* - Give the eligibility criteria, and the sources and methods of case ascertainment and control selection. Give the rationale for the choice of cases and controls  *Cross-sectional study* - Give the eligibility criteria, and the sources and methods of selection of participants  *(b) Cohort study* - For matched studies, give matching criteria and number of exposed and unexposed  *Case-control study* - For matched studies, give matching criteria and the number of controls per case | NA | RECORD 6.1: The methods of study population selection (such as codes or algorithms used to identify subjects) should be listed in detail. If this is not possible, an explanation should be provided.  RECORD 6.2: Any validation studies of the codes or algorithms used to select the population should be referenced. If validation was conducted for this study and not published elsewhere, detailed methods and results should be provided.  RECORD 6.3: If the study involved linkage of databases, consider use of a flow diagram or other graphical display to demonstrate the data linkage process, including the number of individuals with linked data at each stage. | Page 5 GWG data points & variables used in the modelling  Page 5 GWG data points & variables used in the modelling  NA |  |
| Variables | 7 | Clearly define all outcomes, exposures, predictors, potential confounders, and effect modifiers. Give diagnostic criteria, if applicable. | Page 5 *GWG data points & variables used in the modelling* | RECORD 7.1: A complete list of codes and algorithms used to classify exposures, outcomes, confounders, and effect modifiers should be provided. If these cannot be reported, an explanation should be provided. | The exposure being analysed is gestational weight gain (GWG) per gestational week (GA), which is included in the dataset. All other covariates were obtained directly from the dataset and were not derived, except for the GWG at week 0. This information is clearly explained on page 6 under the subheading "GWG Data Points & Variables Used in the Modelling." |  |
| Data sources/ measurement | 8 | For each variable of interest, give sources of data and details of methods of assessment (measurement).  Describe comparability of assessment methods if there is more than one group | The exposure being analysed is gestational weight gain (GWG) per gestational week (GA), which is included in the dataset. All other covariates were obtained directly from the dataset and were not derived, except for the GWG at week 0. This information is clearly explained on page 6 under the subheading "*GWG Data Points & Variables Used in the Modelling."* |  |  |  |
| Bias | 9 | Describe any efforts to address potential sources of bias | The only source of bias would be Clinical measurements of height and weight are considered the gold standard that produces more accurate data on pre-pregnancy BMI than self-reported measurements. We have stated this in the limitation section on page 6 |  |  |  |
| Study size | 10 | Explain how the study size was arrived at | This is a retrospective longitudinal observational study; therefore, a sample size calculation was not performed. |  |  |  |
| Quantitative variables | 11 | Explain how quantitative variables were handled in the analyses. If applicable, describe which groupings were chosen, and why | This is clearly stated in *subsection Statistical analysis* Pages 6-8 |  |  |  |
| Statistical methods | 12 | (a) Describe all statistical methods, including those used to control for confounding  (b) Describe any methods used to examine subgroups and interactions  (c) Explain how missing data were addressed  (d) *Cohort study* - If applicable, explain how loss to follow-up was addressed  *Case-control study* - If applicable, explain how matching of cases and controls was addressed  *Cross-sectional study* - If applicable, describe analytical methods taking account of sampling strategy  (e) Describe any sensitivity analyses | The statistical methods are stated in the subsection *Statistical analysis* Pages 6-8. |  |  |  |
| Data access and cleaning methods |  | .. | Data used in this paper were screened for potential data entry errors. The outcome variable GWG was restricted to four standard deviations from the mean to eliminate implausible values. Page 6. | RECORD 12.1: Authors should describe the extent to which the investigators had access to the database population used to create the study population.  RECORD 12.2: Authors should provide information on the data cleaning methods used in the study. | Data used in this paper were screened for potential data entry errors. The outcome variable GWG was restricted to four standard deviations from the mean to eliminate implausible values. Page 6. |  |
| Linkage |  | .. | NA | RECORD 12.3: State whether the study included person-level, institutional-level, or other data linkage across two or more databases. The methods of linkage and methods of linkage quality evaluation should be provided. | NA |  |
| **Results** | | | | | | |
| Participants | 13 | (a) Report the numbers of individuals at each stage of the study (*e.g.*, numbers potentially eligible, examined for eligibility, confirmed eligible, included in the study, completing follow-up, and analysed)  (b) Give reasons for non-participation at each stage.  (c) Consider use of a flow diagram | This information is provided under Results section, page 8 and 9. | RECORD 13.1: Describe in detail the selection of the persons included in the study (*i.e.,* study population selection) including filtering based on data quality, data availability and linkage. The selection of included persons can be described in the text and/or by means of the study flow diagram. |  |  |
| Descriptive data | 14 | (a) Give characteristics of study participants (*e.g.*, demographic, clinical, social) and information on exposures and potential confounders  (b) Indicate the number of participants with missing data for each variable of interest  (c) *Cohort study* - summarise follow-up time (*e.g.*, average and total amount) | This information is given in Table 1. |  |  |  |
| Outcome data | 15 | *Cohort study* - Report numbers of outcome events or summary measures over time  *Case-control study* - Report numbers in each exposure category, or summary measures of exposure  *Cross-sectional study* - Report numbers of outcome events or summary measures | NA |  |  |  |
| Main results | 16 | (a) Give unadjusted estimates and, if applicable, confounder-adjusted estimates and their precision (e.g., 95% confidence interval). Make clear which confounders were adjusted for and why they were included  (b) Report category boundaries when continuous variables were categorized  (c) If relevant, consider translating estimates of relative risk into absolute risk for a meaningful time period | NA |  |  |  |
| Other analyses | 17 | Report other analyses done—e.g., analyses of subgroups and interactions, and sensitivity analyses | This information is provided results section pages 8-10. |  |  |  |
| **Discussion** | | | | | | |
| Key results | 18 | Summarise key results with reference to study objectives | This information is provided in page10-13 |  |  |  |
| Limitations | 19 | Discuss limitations of the study, taking into account sources of potential bias or imprecision. Discuss both direction and magnitude of any potential bias | This information is provided on page 13 under the subheading strengths and limitations. | RECORD 19.1: Discuss the implications of using data that were not created or collected to answer the specific research question(s). Include discussion of misclassification bias, unmeasured confounding, missing data, and changing eligibility over time, as they pertain to the study being reported. | This information is provided in page10-13 |  |
| Interpretation | 20 | Give a cautious overall interpretation of results considering objectives, limitations, multiplicity of analyses, results from similar studies, and other relevant evidence | This information is provided on page10-13 |  |  |  |
| Generalisability | 21 | Discuss the generalisability (external validity) of the study results | This information can be found on pages 10 to 13. |  |  |  |
| **Other Information** | | | | | | |
| Funding | 22 | Give the source of funding and the role of the funders for the present study and, if applicable, for the original study on which the present article is based | NA |  |  |  |
| Accessibility of protocol, raw data, and programming code |  | .. |  | RECORD 22.1: Authors should provide information on how to access any supplemental information such as the study protocol, raw data, or programming code. | This is included on page 15 |  |

*Reference: Benchimol EI, Smeeth L, Guttmann A, Harron K, Moher D, Petersen I, Sørensen HT, von Elm E, Langan SM, the RECORD Working Committee. The REporting of studies Conducted using Observational Routinely-collected health Data (RECORD) Statement. *PLoS Medicine* 2015; in press.

*Checklist is protected under Creative Commons Attribution ([CC BY](http://creativecommons.org/licenses/by/4.0/)) license.
