# Supplementary material for: Comparison of statistical modelling methods for population-level gestational weight gain trajectories in ethnically diverse women in southeast Melbourne, Australia
Source: BMJ Open. 2025 Mar 13;15(3):e088664. doi: 10.1136/bmjopen-2024-088664 (PMC11906984; doi:10.1136/bmjopen-2024-088664)
Supplement: Supplementary file 2 [file bmjopen-2024-088664.reviewer_comments.pdf]

PEER REVIEW HISTORY

BMJ Open publishes all reviews undertaken for accepted manuscripts. Reviewers are asked to complete a checklist review form and are provided with free text boxes to elaborate on their assessment. These free text comments are reproduced below.

ARTICLE DETAILS

Title (Provisional)

Comparison of statistical modelling methods for population level gestational weight gain trajectories in ethnically diverse women in south east Melbourne, Australia

Authors

Ranasinha, Sanjeeva; Teede, Helena J.; Harrison, Cheryce; Wang, Rui; Enticott, Joanne

---

VERSION 1 - REVIEW

**Reviewer**	**1**
**Name**	**Yamada, Goro**
**Affiliation**	**Drexel University, Dornsife School of Public Health**
**Date**	**08-Sep-2024**
**COI**	**None**

Different modeling methods for gestational weight gain (GWG) were assessed. The model descriptions and results presentations require improvement. There are many assessed models, and I would recommend narrowing down candidate models (although the authors might have assessed many of them) for the presentation in a manuscript.

Introduction, the last paragraph:

- Modeling GEG: Women change their weight-related behaviors during pregnancy influenced by the attending physicians and others. Can they be considered in the model?

Method, Study design and setting:

- [Minor] We should be used instead of I? There are multiple authors for this manuscript.

- I could not understand why the bias is a concern in relation to race and ethnicity. Could the reasons be elaborated?

Participants

- "As women had multiple measurements… to conduct analysis" is unnecessary.

Statistical analysis, GWG data points & variables used in the modeling

- Why are ethnic-specific BMI categories used only for Chinese and Koreans? Are they different in body structures, or is BMI categorization different in these countries? How about other Asian descendants? I recommend using the same category and adjusting for race-ethnicity in the following analysis.

- “We modelled GWG by gestational age, parity, ethnicity, and age.” Specify which variables were included as continuous and categorical. If categorical, specify the number of categories or dummy variables. State if they were time-invariant or time-fixed variables.

- State how the correlation of longitudinal measurements was incorporated into the analyses.

- “BMI as a continuous variable, as categorizing BMI can result in information loss”: If this is the case, why was ethnic-specific BMI mentioned previously??

- Is it an option to drop data at week zero or use weight, not GWG, as an outcome with the longitudinal data analysis method? It is obvious that GWG is zeto at week 0.

Baseline weight reliability – aim 1

- Show model equations for both two-way mixed effects models and GAMLSS models as supplemental material. Describe why the neural network method was considered.

- Explain how GAMLESS models can be applied to measure baseline weight reliability. What is meant by “compare the trajectories of self-reported weight with weight measured at the first visit”? Comparing trajectories among study subjects?

Optimal GWG modeling – aim 2

- The same comment as the previous one. Show model equations for all the models used.

- For the GAMLSS models, the authors tried three smoothing techniques and several distributions. There are so many models in the paper. I think models should be selected for the presentation in this paper.

- About the measurement of goodness of fit. I wonder if the authors have checked if the predicted lines fit well with individual GWG trajectories.

Sensitivity analysis

- State how trajectories were compared.

Results

Aim 1

- What is meant by the consistency of agreement? Weight is a continuous variable. State how the consistency was measured.

Aim 2

- Include the used sample size somewhere in the result section. I see a description in the abstract but not elsewhere. It is informative to inform the range of the number of records per person (e.g., min, max, and median).

- Present the summary statistics and graphical summaries in a way so that readers can compare models.

Figure 1

- Graph C is the same as B. Are the mother's age and BMI the pre-pregnancy measures? If so, how was GWG measured? (Graphs B-E) Last measurement – baseline? However, the last measurements vary across mothers in timing (e.g., close to the point of delivery or several weeks before).

- Graph F. The authors state that the GAMLSS model was superior, but this graph comes from the generalized additive model. Why? Also, state how included covariates were fixed, or is this a model with gestational week as the only covariate?

- The description of confidence intervals should be given in the figures.

Figure 3.

- If the weight measurement at the first visit is used for modeling, how can trajectories before the first measurement (e.g., weeks 0-4) be estimated?

Figure 2

- How were these four graphs obtained if the BMI was included as a continuous variable in the model?

Supplemental data

- Figure S1-S5: Show the same statistics and graphs across different models. What do mean and variable presents?

- Figure S2. I do not understand this graph. Explain this model in the method section.

- Figure S6. State clearly what is presented in the y-axis. Present graphs appropriate for publication.

- Supplemental material: The method section says several distributions were assessed, but it is not clearly stated.

Discussion

- Describe why the GAMLSS method is better than others. Is using the same covariates for mu, sigma, nu, and tau necessary? I would like to see the authors' evaluation of different GAMLSS models – which smoothing function is better, and which distribution is better?

- What are the advantages of using model-based values instead of observed values in the centile graphs?

**Reviewer**	**2**
**Name**	**Hailegebireal, Aklilu**
**Affiliation**	**Auckland University of Technology, FHES**
**Date**	**20-Oct-2024**
**COI**	**no**

I am pleased to provide my comments on the manuscript titled "Comparison of Statistical Modelling Methods for Population-Level Gestational Weight Gain Trajectories in Ethnically Diverse Women: A Longitudinal Observational Study."

This manuscript is well-written and offers significant insights into the field of epidemiological studies, particularly those focusing on gestational weight gain (GWG). The comprehensive analysis and comparison of statistical modelling methods presented in this study have the potential to greatly enhance our understanding of GWG trajectories across diverse populations, making it a valuable contribution to future research and public health initiatives.

By saying this I will try to forward my possible comments and suggestions on this piece of work.

Abstract

• Method section: Despite the authors mentioned the models that were utilized by previous studies, they didn't clearly showed their means of analysis to came up with their results

• Results: It would be better to mention the second-best model to give more full information for the readers. 'The best model is GAMLSS with generalised R-squared of 0.70 followed by…'

• Conclusion: Make it straightforward in a way that is easy to understand and more actionable

Strength and limitation

• Make them more short and precise in a way that is easily understandable to the readers.

Introduction

• Line three of the first paragraph 'health GWG… I think it means Healthy GWG'

• It would be better to have a paragraph that deals with GWG(normal vs abnormal) as per IOS…

• Most of the statements were not supported by references

• There are plenty of deep statistical terminologies like… skewed and kurtotic… in the introduction section and better to define them in alternative ways

• In the last paragraph, it says 'The end goal of this project is to develop a pragmatic and a robust algorithm to underpin an application that could be widely used in a clinical setting to reliably estimate GWG for each pregnant person.' Thus, the most robust algorithm has to be mentioned in the result section of the abstract

Methods

General comment: despite it being comprised of various statistical concepts, it was not well written and there are plenty of vague statements difficult to comprehend. Thus, it needs proofreading.

• 'To avoid any type of bias…' This might be statistically unsound and better to state as 'to minimize possible biases'

• Better to report in general rather than directing those activities to the first person. For instance, '…I used the Prediction model….'

• There are lengthy sentences that need to be synthesized. For instance 'To avoid any form of bias when including ethnicity in my analyses, I used the Prediction model Risk of Bias Assessment Tool (PROBAST) and to evaluate of risk-of bias specific to the inclusion of race and ethnicity in my prediction model, Critical Appraisal for Racial and Ethnic Equity in Clinical Prediction Models (CARE-CPM) which builds upon the existing PROBAST tool.'…..this should be well narrated in its simplest form

• I couldn't find any statement about the eligibility criteria (inclusion and exclusion criteria with their possible justifications). Why multiple pregnancies were excluded?....

• There is a lack of detailed description of the data that were used for this study

• Using different means of categorization for GWG(WHO, ethnic-specific BMI categories for Chinese and Korean BMI categories …) might cause the issue of generalizability…

• 'Birth weight ???' is that maternal weight at childbirth, after childbirth, of the newborn weight…..so, make it clear!

• How the covariates parity and ethnicity could be continuous variables? (they are categorical ones)

• Grammatically, plenty of subject disagreements: I, we,…

• Some statements in the method section seem results rather than showing the actual methodological activities.

• The software used was not mentioned

Results

• At the entry point, there should be a detailed explanation of the background characteristics of the study participants

• It's difficult to capture the basic findings since the author refers to the appendix for every result. Thus I suggest reporting basic findings first and then referring to supporting documents for the details

---

VERSION 1 - AUTHOR RESPONSE

**BMJ's research committee meeting: Comments**	Author response to the comments	Additional information
**Reviewer 1 (Dr. Goro Yamada)**	Thank you very much for your valuable comments and suggestions.	
Different modeling methods for gestational weight gain (GWG) were assessed. The model descriptions and results presentations require improvement. There are many assessed models, and I would recommend narrowing down candidate models (although the authors might have assessed many of them) for the presentation in a manuscript.	This is an excellent recommendation for the structure of the paper, which we have now done. This includes this new paragraph added into the methods section:<br><br>"Optimal GWG modelling – aim 2;<br>Our aim was to determine the optimal method to model GWG trajectories in women across gestational age adjusting for significant independent variables listed above that are known to influence GWG trajectories. As GWG trajectories by gestation time appeared non-linear; we examined several statistical modelling approaches to determine which method fits GWG data the best. Multiple models were examined (7 in total), and for brevity only four of these will be included in detail in this manuscript (**others included in Supplementary files**). Three of these models will be the best performing models, and another will be the linear regression model as a baseline model for comparison and to facilitate the interpretation of the coefficients. The three best performing models were: Cubic Polynomial, and two generalised additive model for location, scale, and shape (GAMLSS) Box-Cox model with original distribution and with different smoothing functions (one with cubic splines10 and another with penalised B splines11)."	
Modeling GEG: Women change their weight-related behaviors during pregnancy influenced by the attending physicians and others. Can they be considered in the model?	We could not include this information as this data was not available to us. We have added this into the discussion section within the limitations paragraph:<br>"As this is routine health data, we were unable to examine if women change their weight-related behaviours during pregnancy influenced by the attending physicians and others; however, we aim to examine this prospectively in a pragmatic trial using this model to predict GWG"	
Method, Study design and setting:	The word "we" is now used throughout the manuscript. We have now corrected the usage of "I" to "we" in the supplementary files.	

We should be used instead of I? There are multiple authors for this manuscript.		
I could not understand why the bias is a concern in relation to race and ethnicity. Could the reasons be elaborated?	This statement has been removed from the manuscript.	
As women had multiple measurements… to conduct analysis" is unnecessary.	We removed this statement.	
Why are ethnic-specific BMI categories used only for Chinese and Koreans? Are they different in body structures, or is BMI categorization different in these countries? How about other Asian descendants? I recommend using the same category and adjusting for race-ethnicity in the following analysis.	This is true, however the only documented information (Journal articles) available to us were for Chinese and Korean women. There are no reliable journal articles that have used cut-off points for other ethnicities. This work is underway and can be dynamically incorporated into the algorithm as they are generated and validated.	
We modelled GWG by gestational age, parity, ethnicity, and age." Specify which variables were included as continuous and categorical. If categorical, specify the number of categories or dummy variables. State if they were time-invariant or time-fixed variables.	This information has been added into the manuscript in the methods section in the first paragraph under the Statistical analysis heading; "Maternal pre-pregnancy BMI and age were continuous variables. Ethnicity was determined by country of birth, and we categorised ethnicity into 12 regions defined by Australian Bureau of Statistics (supplemental Table S5). Parity was captured in 3 categories (0, 1-3, >3). They were time fixed variables. Gestational age and GWG were continuous variables and varied with time."<br>Please also see table one for more details.	
State how the correlation of longitudinal measurements was incorporated into the analyses.	The longitudinal model GAMLSS incorporates the correlation of the measurements through smoothing techniques. We used penalised B -spline smoother which is the recommended method by the authors [1]. Please see supplementary material in the Appendix pages 10-11.	[1] Stasinopoulos DM, Rigby RA. Generalized Additive Models for Location Scale and Shape (GAMLSS) in R. J. Stat. Soft. [Internet]. 2007 Dec. 31 [cited 2025 Jan. 9];23(7):1-46. Available from: https://www.jstatsoft.org/index.php/jss/article/view/v023i07
BMI as a continuous variable, as categorizing BMI can result in information loss": If this is the case, why was ethnic-specific BMI mentioned previously?	We used BMI as a continuous variable in building the model, therefore, the continuous BMI variable is an independent variable. However, a normal BMI for one ethnic group, might be abnormal in another ethic group. Therefore, we created different subsets of data based on these BMI categories. We then generated GWG trajectories using the model for each of these subsets.<br>Then we could see the GWG trajectory in women with a normal pre-pregnancy BMI despite their ethnicity.<br>See the GWG for the four BMI categories separately as shown in Figure 3. We did this because our clinician stakeholders and researchers want to see it. The different BMI categories are known to be associated with different clinical outcomes. This has been added into the methods section under the heading for Feasibility of GWG trajectory centile graphs- aim 3:	

	"We will also generate four separate trajectory centile charts, one for each of the pre-pregnancy BMI categories. These latter outputs were requested by our clinician stakeholders and researchers because the different BMI categories are known to be associated with different clinical outcomes".	
Is it an option to drop data at week zero or use weight, not GWG, as an outcome with the longitudinal data analysis method? It is obvious that GWG is zero at week 0.	It is an option to drop data at week zero, and we did this in the sensitivity analysis (instead using the weight at first measurement as the new baseline). To avoid the having a 'zero' value at week zero, we instead replaced it with a random draw from the gaussian distribution and since the measurement error measurement error of a single dial measurement is about 0.70 kg, so the variance of the gain score is equal to $0.70^2 + 0.70^2 = 0.98$ kg. For each woman, the weight gain at the start of pregnancy was taken as a random draw from the Gaussian distribution with mean of 0 and variance of 0.98 kg. The size of the measurement error was used since it is theoretically based but any variance could have been applied. This could be considered a nudge to initiate the iterations. If we took the first measurement as starting point it would have introduced instability as fourteen percent (5713/39846) of the measurements would have been staring measurements. Please see figure 2 Page 7.	
Baseline weight reliability – aim 1<br>Show model equations for both two-way mixed effects models and GAMLSS models as supplemental material. Describe why the neural network method was considered.	Model equations are shown in the supplementary material in the Appendix pages 10-11. Model equations for the two-way mixed effects models are the standard ones (we can include this in the Appendix if required).<br>We have added this sentence into the methods: "We also examined a neural network method to preliminary explore if a machine learning technique could outperform conventional methods."	
Explain how GAMLESS models can be applied to measure baseline weight reliability. What is meant by "compare the trajectories of self-reported weight with weight measured at the first visit"? Comparing trajectories among study subjects?	We plotted the trajectories using week 0 (gaussian distribution) as starting values and the measurement at first visit. Please see the new caption for figure 2 for the description.	
Optimal GWG modeling – aim 2<br>The same comment as the previous one. Show model equations for all the models used.	Please see supplementary material in the Appendix pages 10-11.	
For the GAMLSS models, the authors tried three smoothing techniques and several distributions. There are so many models in the paper. I think models should be selected for the presentation in this paper.	We agree with the reviewer and have restructured the manuscript accordingly. See response above to the first comment by this reviewer.	
About the measurement of goodness of fit. I wonder if the authors have checked if	Yes, we did. We used the goodness of fit statistics to evaluate model performance, we compared model indices and residual statistics by visual inspection of quantile-	

the predicted lines fit well with individual GWG trajectories.	quantile plots of the residuals, plots of residuals versus fitted values, detrended quantile-quantile plots and worm plots (Appendix Figure S6). Goodness-of-fit was evaluated using Akaike information criteria (AIC) and Schwarz/Bayesian information criteria (SBIC). We conducted an analysis of gestational weight gain trajectories for the observed data and the trajectories derived from the Generalised Additive Models for Location Scale and Shape (GAMLSS) model for the four BMI categories for our second paper "Gestational weight gain charts for different body mass index groups and adherence to NAM guidelines in ethnically diverse women." This analysis indicated good model fit (see the Figure below).	
Sensitivity analysis<br>State how trajectories were compared.	To confirm aim1 results, the fourteen percent (5713/39846) of the original dataset with weights measured at the first antenatal visit were analysed on a sensitivity analysis, where these weight measurements become the new baseline. Overlayed on the trajectory centile charts (solid lines; Figure 2) are centile charts determined by the sensitivity analysis data set (dotted lines; Figure 2). Before 20 weeks, comparisons are difficult to make due to a high proportion of measurements in the sensitivity analysis now becoming the new baseline (i.e., 76% of first visit weight measurements were in the 1–20-week period). However, beyond 25 weeks gestation, the trajectories from the two models converge, which indicates that the baseline for measuring GWG (prepregancy or first antenatal visit) had little effect on the gestation trajectories in the latter pregnancy stages. See the new caption for figure 2.	
Results Aim 1<br>What is meant by the consistency of agreement? Weight is a continuous variable. State how the consistency was measured.	We used ICC to test the consistency.<br>To assess the reliability of baseline weight we used two-way mixed effects models to calculate the intraclass correlation coefficient (ICC) using an absolute agreement definition and the consistency of agreement. This means we evaluate whether the two data points (self-reported weight at the start of pregnancy and weight at the first measurement) match each other exactly. If they do not match exactly, we also check if one measurement is consistently higher or lower than the other.	
Include the used sample size somewhere in the result section. I see a description in the abstract but not elsewhere. It is informative to inform the range of the number of records per person (e.g., min, max, and median).	The number of GWG measurements for each woman was inserted in the manuscript (page 5). Directly under the results heading we now include this paragraph:<br>"Data included 39,846 women, with 109,339 GWG measurements. The average number of GWG measurements for each woman was 2.85 (min=1 and max=20). Table 1 shows the characteristics of the pregnant sample (n =39846)"	
Present the summary statistics and graphical summaries in a way so that readers can compare models.	The model comparisons are now provided in table 2.	
Figure 1<br>Graph C is the same as B. Are the mother's age and BMI the pre-pregnancy measures? If so, how was GWG measured?	Thank you, now it is fixed. Please see table 4.<br>Yes, the baseline mothers age and BMI are pre-pregnancy. Modeling was done using pairs of data (GWG, gestion week), where the change since pre-pregnancy weight at the specified gestation in weeks. This means that we can model	

(Graphs B-E) Last measurement – baseline? However, the last measurements vary across mothers in timing (e.g., close to the point of delivery or several weeks before)	the trajectory (GWG vs time) and it doesn't matter if women have their GWG measured at differing time points.	
Graph F. The authors state that the GAMLSS model was superior, but this graph comes from the generalized additive model. Why? Also, state how included covariates were fixed, or is this a model with gestational week as the only covariate?<br>- The description of confidence intervals should be given in the figures.	Graph F is included only to show the non-linearity of trajectories in GWG (and the non-linearity is similar regardless of whether GAM or GAMLSS was used. This information has now been added into the caption i.e. " Graph F shows the smoothed GWG(Δ) by gestational age (weeks) from a univariate generalised additive model (included to show the non-linearity of trajectories in GWG. We also included description of the 95% CI as suggested by the reviewer. Note: similar pattern exhibited when other non-linear modelling applied)."	
Figure 3<br>If the weight measurement at the first visit is used for modeling, how can trajectories before the first measurement (e.g., weeks 0-4) be estimated?	Figure 2 shows the GWG trajectories generated when including the self-reported pre-pregnancy weight at week zero (solid lines). Dotted lines show the GWG trajectories generated when the measured weight at first visit is used as the week zero weight instead. To avoid using a 'zero' GWG value, both week zero distributions These were estimated using a Gaussian distribution. As mentioned previously, and also added into the caption for Figure 2.<br><br>The measurement error of a single dial measurement is about 0.70 kg, so the variance of the gain score is equal to $0.70^2 + 0.70^2 = 0.98$ kg. For each woman, the weight gain at the start of pregnancy was taken as a random draw from the Gaussian distribution with mean of 0 and variance of 0.98 kg. The size of the measurement error was used since it is theoretically based but any variance could have been applied [2]. This could be considered a nudge to initiate the iterations [3]. Figure 2 shows the GWG trajectories generated when including the self-reported pre-pregnancy weight at week zero (solid lines). Dotted lines show the GWG trajectories generated when the measured weight at first visit is used as the week zero weight instead. To avoid using a 'zero' GWG value, both week zero distributions were estimated using a Gaussian distribution. As mentioned previously and added into the caption for Figure 2.	[2] Yorkin M, Spaccarotella K, Martin-Biggers J, Quick V, Byrd-Bredbenner C. Accuracy and consistency of weights provided by home bathroom scales. BMC Public Health 2013;13:1194.<br><br>[3] Santos S, Eekhout I, Voerman E, et al. Gestational weight gain charts for different body mass index groups for women in Europe, North America, and Oceania. BMC Med 2018;16:201.
Figure 2<br>How were these four graphs obtained if the BMI was included as a continuous variable in the model?	See above: We used BMI as a continuous variable in building the model, therefore, the continuous BMI variable is an independent variable. However, a normal BMI for one ethnic group, might be abnormal in another ethic group. Therefore, we created different subsets of data based on these BMI categories. We then generated GWG trajectories using the model for each of these subsets.<br>Then we could see the GWG trajectory in women with a normal pre-pregnancy BMI despite their ethnicity.<br>See the GWG for the four BMI categories separately as shown in Figure 3. We did this because our clinician stakeholders and researchers want to see it. The different BMI categories are known to be associated with different clinical outcomes.	

Figure S1-S5: Show the same statistics and graphs across different models. What do mean and variable presents?	The mean statistic represents summary of the randomised quantile residuals. The closer this statistic is to zero the better the model.	
Figure S2. I do not understand this graph. Explain this model in the method section.	In Figure S2, the lines connecting the neurons are the weights between them where black lines represent positive weights, and grey lines represent negative weights. The thickness of the lines specifies the strength the weight: thick lines have a greater absolute value than thin lines. Each of the 17 random variables are shown in the first layer (labelled by name) and the response variable is shown in the far-right layer (labelled as ‘$g_1(_\mu)$. The hidden layer is labelled as H1 through H5, which was specified using the size argument in the nnet function. B1 and B2 are bias layers that apply constant values to the nodes, similar to intercept terms in a regression model.	
Figure S6. State clearly what is presented in the y-axis. Present graphs appropriate for publication.	This plot is the plot of the fitted terms for model Box Cox t-original distribution The Y axis is the partial of the smoothing technique for the variable we are testing in the case it was P-spline smoother (pb), We have modified the graph as suggested to make it more presentable.	
Supplemental material: The method section says several distributions were assessed, but it is not clearly stated.	We have restructured the paper to present this information more clearly.	
Discussion<br>Describe why the GAMLSS method is better than others. Is using the same covariates for mu, sigma, nu, and tau necessary? I would like to see the authors' evaluation of different GAMLSS models – which smoothing function is better, and which distribution is better?<br>- What are the advantages of using model-based values instead of observed values in the centile graphs?	Added into the discussion are these new text:<br>“Overall, we propose that the GAMLSS model is optimal for managing complex GWG data, especially as the residual plots for the other models showed non-normality and skewness (Figure 1 and, Figure S1). The main characteristic of GAMLSS models is the ability to allow the location, scale and shape of the distribution of the response variable to vary according to the values of explanatory variables. Additionally, it is semiparametric, which offers several advantages including that it can use a wider range of distributions, including the distribution of the exponential family and other distributions, and in fact, our two best models were GAMLSS: one fitted with penalised B splines and the other with cubic splines.”<br><br>“Using the optimal model, we demonstrate how to generate centile charts for the sample population. While our sample size was large and observed values can also produce centile graphs, the key advantage of using model-based values is their ability to create more personalized and representative centiles tailored to an individual’s characteristics, as model input factors are maternal age, pre-pregnancy BMI, parity, and ethnicity (country of birth). Additionally, model-based values enable the creation of centile charts even when data points are limited, thus offering the potential for more personalized charts that can be applied to a broader range of individuals.”	

Figure. Gestational weight gain trajectories for the observed data and the trajectories derived from the optimal model (by the four BMI categories). Note this figure is included for the reviewers only. It is included in another paper currently under review. Therefore it wasn't included in this manuscript.

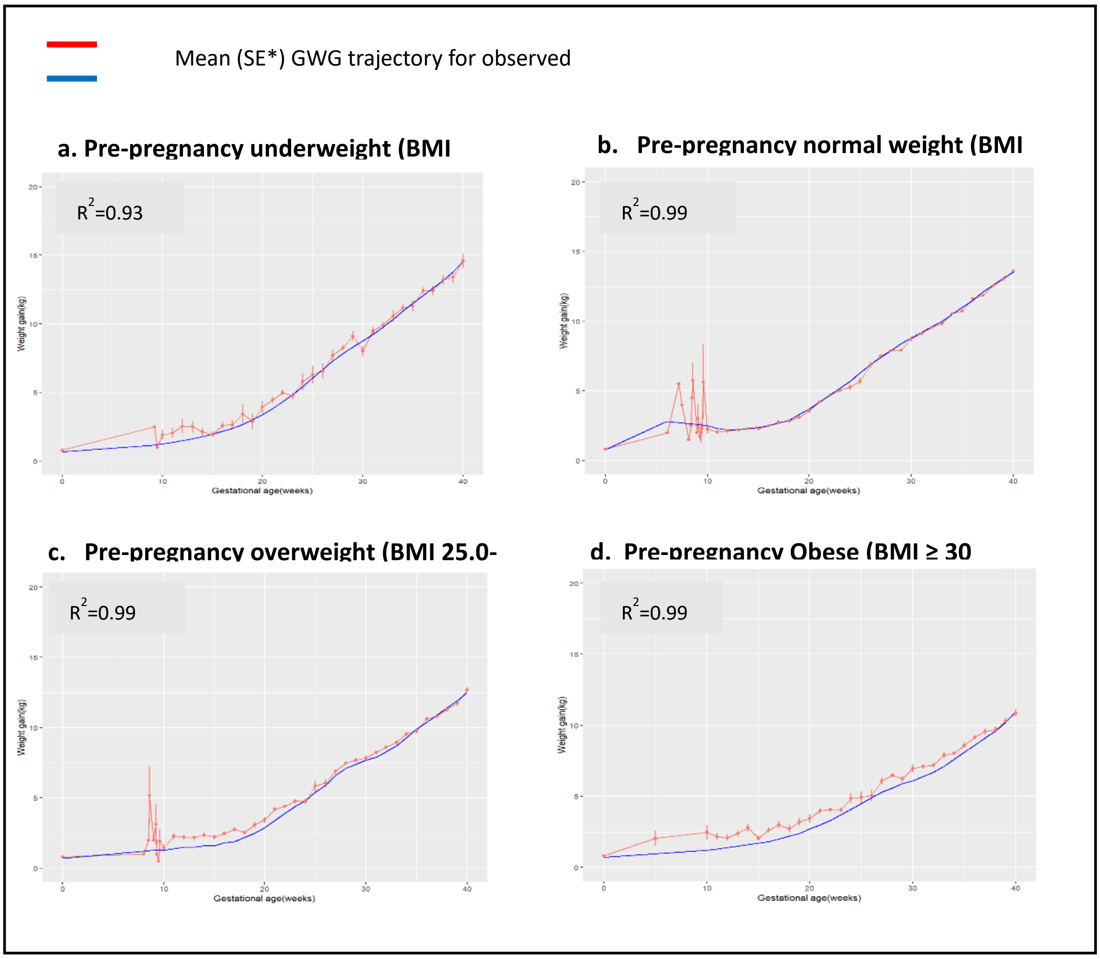

Reviewer 2 (Dr. Aklilu Hailegebireal)		
This manuscript is well-written and offers significant insights into the field of epidemiological studies, particularly those focusing on gestational weight gain (GWG). The comprehensive analysis and comparison of statistical modelling methods presented in this study have the potential to greatly enhance our understanding of GWG trajectories across diverse populations, making it a valuable contribution to future research and public health initiatives.<br>By saying this I will try to forward my possible comments and suggestions on	Thank you very much for your valuable comments and suggestions.	

this piece of work.		
Abstract:<br>Method section: Despite the authors mentioned the models that were utilized by previous studies, they didn't clearly showed their means of analysis to came up with their results.	This is now rectified.	
Results: It would be better to mention the second-best model to give more full information for the readers. 'The best model is GAMLSS with generalised R-squared of 0.70 followed by…'	This is now done.	
Conclusion: Make it straightforward in a way that is easy to understand and more actionable	This is now done.	
Strength and limitation<br>Make them shorter and more precise in a way that is easily understandable to the readers.	This is now done.	
Introduction<br>Line three of the first paragraph 'health GWG… I think it means Healthy GWG	Thank you we changed it.	
It would be better to have a paragraph that deals with GWG(normal vs abnormal) as per IOS	As requested, this the new paragraph inserted into the introduction:<br>As GWG is a critical factor in maternal and fetal health, the Institute of Medicine (IOM) have provided guidelines based on pre-pregnancy BMI1. These BMI guides are based on the World Health Organization BMI classifications, which were primarily derived from studies in predominantly White, Western populations1. BMI categories can be different for different ethnic groups; and in the published literature are BMI categories for Chinese and Korean populations, with more ethic	

	specific data forthcoming2,15-17. GWG deviations from the IOM recommendations—either excessive or inadequate weight gain—are associated with adverse outcomes1,2,3. Excessive GWG increases the risk of gestational diabetes, hypertensive disorders, cesarean delivery, and childhood obesity, while inadequate GWG raises the likelihood of preterm birth, low birth weight, and fetal growth restriction1,2,3. IOM recommend monitoring and individualized guidance are essential to optimize maternal and neonatal outcomes1,2,3.	
Most of the statements were not supported by references	This has been rectified.	
There are plenty of deep statistical terminologies like… skewed and kurtotic… in the introduction section and better to define them in alternative ways	In the introduction, expanded meanings for skewed and kurtotic are now provided.	
In the last paragraph, it says 'The end goal of this project is to develop a pragmatic and a robust algorithm to underpin an application that could be widely used in a clinical setting to reliably estimate GWG for each pregnant person.' Thus, the most robust algorithm has to be mentioned in the result section of the abstract	This is now done.	
Method<br>General comment: despite it being comprised of various statistical concepts, it was not well written and there are plenty of vague	This has now been extensively proofread by the multidisciplinary author group.	

statements difficult to comprehend. Thus, it needs proofreading.<br><br>To avoid any type of bias…' This might be statistically unsound and better to state as 'to minimize possible biases'	We hope it reads to a better standard now.<br><br><br><br>Thank you this is corrected	
Better to report in general rather than directing those activities to the first person. For instance, '…I used the Prediction model	This is fixed, thank you.	
There are lengthy sentences that need to be synthesized. For instance 'To avoid any form of bias when including ethnicity in my analyses, I used the Prediction model Risk of Bias Assessment Tool (PROBAST) and to evaluate of risk-of bias specific to the inclusion of race and ethnicity in my prediction model, Critical Appraisal for Racial and Ethnic Equity in Clinical Prediction Models (CARE-CPM) which builds upon the existing PROBAST tool.'…..this should be well narrated in its simplest form	This is fixed, thank you	
I couldn't find any statement about the eligibility criteria (inclusion and exclusion criteria with their possible justifications). Why multiple pregnancies were excluded?.	As this was an observational study, we did not set out exclusion/inclusion criteria. Yet multiple pregnancies were excluded because the GWG trajectory is expected to be different to singleton pregnancies. This last point is now added into the methods Please see page 3.	
There is a lack of detailed description of the data that were used for this study	We have now added a paragraph directly under the results heading to describe the data.	
Using different means of categorization for GWG(WHO, ethnic-specific BMI categories for Chinese and Korean BMI categories …) might cause the issue of generalizability	We agree that the results are likely to be more generalizable due to the diverse sample and different means to categorise the GWG.	
Birth weight ???' is that maternal weight at childbirth, after childbirth, of the newborn weight…..so, make it clear!	This is the birthweight of the newborn baby. We corrected this in the manuscript.	
How the covariates parity and ethnicity could be continuous variables? (they are categorical ones)	Yes, this is correct they are categorical. We changed the manuscript to make this clear.	
Grammatically, plenty of subject disagreements: I, we,	This is fixed now thank you.	
Some statements in the method section seem results rather than showing the actual methodological activities.	There are some percentiles and their corresponding standard deviations mentioned which we believe is necessary in this case to understand the method of centile creation.	

The software used was not mentioned	Software is added "Moreover, we assessed several distributions using the GAMLSS package in R version 4.1.2" (Page 4)	
REsults<br>At the entry point, there should be a detailed explanation of the background characteristics of the study participants	We have now added a paragraph directly under the results heading to describe the data.	
It's difficult to capture the basic findings since the author refers to the appendix for every result. Thus I suggest reporting basic findings first and then referring to supporting documents for the details	We agree. Therefore, we have followed the recommendation by reviewer 1 recommendation for the structure of the paper, which we have now done. This includes this new paragraph added into the methods section:<br>"Optimal GWG modelling – aim 2;<br>Our aim was to determine the optimal method to model GWG trajectories in women across gestational age adjusting for significant independent variables listed above that are known to influence GWG trajectories. As GWG trajectories by gestation time appeared non-linear; we examined several statistical modelling approaches to determine which method fits GWG data the best. Multiple models were examined (7 in total), and for brevity only four of these will be included in detail in this manuscript (others included in Supplementary files). Three of these models will be the best performing models, and another will be the linear regression model as a baseline model for comparison and to facilitate the interpretation of the coefficients. The three best performing models were: Cubic Polynomial, and two generalised additive model for location, scale, and shape (GAMLSS) Box-Cox model with original distribution and with different smoothing functions (one with cubic splines10 and another with penalised B splines11)."	
